# Viscoplastic Couette Flow in the Presence of Wall Slip with Non-Zero Slip Yield Stress

**DOI:** 10.3390/ma12213574

**Published:** 2019-10-31

**Authors:** Yiolanda Damianou, Pandelitsa Panaseti, Georgios C. Georgiou

**Affiliations:** Department of Mathematics and Statistics, University of Cyprus, Nicosia 1678, Cyprus; yiolandadamianou@hotmail.com (Y.D.); pandelitsa_@hotmail.com (P.P.)

**Keywords:** yield stress fluids, Bingham plastic, slip, slip yield stress, circular Couette flow, rheometry

## Abstract

The steady-state Couette flow of a yield-stress material obeying the Bingham-plastic constitutive equation is analyzed assuming that slip occurs when the wall shear stress exceeds a threshold value, the slip (or sliding) yield stress. The case of Navier slip (zero slip yield stress) is studied first in order to facilitate the analysis and the discussion of the results. The different flow regimes that arise depending on the relative values of the yield stress and the slip yield stress are identified and the various critical angular velocities defining those regimes are determined. Analytical solutions for all the regimes are presented and the implications for this important rheometric flow are discussed.

## 1. Introduction

Yield-stress, or viscoplastic, materials constitute a very important class, which includes foams, emulsions, colloids, gels, pastes, and suspensions that are of great interest in pharmaceutics, cosmetics, food, oil, and construction industries [1]. These materials behave like fluids when the stress exceeds a certain critical stress, the yield stress, $\tau_{0}^{*}$; otherwise, they behave like solids [2]. It should be noted that throughout the paper symbols with stars denote dimensional variables. 

The Bingham-plastic model is the simplest constitutive equation describing viscoplastic behavior. If $\tau^{*}$ is the viscous stress tensor, ${\dot{\gamma }}^{*}\equiv \nabla u^{*}+{\left(\nabla u^{*}\right)}^{T}$ is the rate-of-strain tensor, where $u^{*}$ is the velocity vector and the superscript *T* denotes the transpose, and $\tau^{*}\equiv \sqrt{\tau^{*}:\tau^{*}/2}$ and ${\dot{\gamma }}^{*}\equiv \sqrt{{\dot{\gamma }}^{*}:{\dot{\gamma }}^{*}/2}$ are the magnitudes of $\tau^{*}$ and ${\dot{\gamma }}^{*}$, respectively, then the Bingham-plastic model can be written as follows:

(1) $$\{\begin{array}{ll} {\dot{\gamma }}^{*}=0, & \tau^{*}\leq \tau_{0}^{*}\\ \tau^{*}=\left(\frac{\tau_{0}^{*}}{{\dot{\gamma }}^{*}}+\mu^{*}\;\right){\dot{\gamma }}^{*}, & \tau^{*}>\tau_{0}^{*} \end{array},$$

where $\mu^{*}$ is the plastic viscosity. Other popular viscoplastic constitutive equations are the Casson and Herschel-Bulkley models, which are able to describe post-yield shear thinning or shear thickening [3]. It is clear that the flow domain in a viscoplastic flow consists of yielded ($\tau^{*}>\tau_{0}^{*}$) and unyielded ($\tau^{*}<\tau_{0}^{*}$) regions, separated by the yield surfaces where $\tau^{*}=\tau_{0}^{*}$. The determination of these regions is not a trivial task, especially in two- and three-dimensional flows [4]. 

Yield-stress materials are also known to exhibit wall slip [5,6]. As pointed out by Hatzikiriakos [7], it appears that slip is the rule and not the exception in several classes of complex fluids, especially viscoplastic ones, such as microgels, glasses, suspensions and pastes. The role of wall slip in various processes of industrial importance has been emphasized in many review papers and experimental studies ([6,7,8,9,10,11]; and references therein). In general, slip may be due to loss of adhesion of the fluid to the wall (true slip) or to the formation of a thin layer adjacent to the wall, where the viscosity is much smaller than in the bulk. The causes and mechanisms of slip in complex fluid flows and the various factors affecting slip have been reviewed in [7,11]. 

Experimental data on different fluid systems, including Newtonian liquids, polymer melts, gels, and suspensions have shown that wall slip occurs only above a certain critical value of the wall shear stress, known as the slip or sliding or threshold yield stress, $\tau_{c}^{*}$ [11,12,13,14,15,16]. The following two-branch equation was proposed for hard-sphere colloidal suspensions [17,18,19,20] and carbopol gels [21]:

(2) $$u_{w}^{*}=\{\begin{array}{ll} 0, & \tau_{w}^{*}\leq \tau_{c}^{*}\\ \frac{{\left(\tau_{w}^{*}-\tau_{c}^{*}\right)}^{s}}{\beta^{*}}, & \tau_{w}^{*}>\tau_{c}^{*} \end{array},$$

where $\tau_{w}^{*}$ is the wall shear stress, $u_{w}^{*}$ is the slip velocity, defined as the relative velocity of the fluid respect to that of the wall, $\beta^{*}$ is the slip coefficient, and s is the exponent. Sochi [14] pointed out that $\tau_{c}^{*}$ characterizes the fluid-solid system and the existing physical conditions. For a fluid with given molecular parameters, the slip coefficient $\beta^{*}$ depends on the temperature, the normal stress and pressure, and on the properties of the fluid/wall interface. In the absence of slip, $\beta^{*}$ is infinite. 

In the case of concentrated suspensions, it was found that s=1 for hydrophilic (repulsive) surfaces when $\tau^{*}<\tau_{0}^{*}$ and s=2 for hydrophobic (attractive) surfaces for a wider range of applied shear stress [11]. Equation (2) has also been used to describe strong slip of polymer melts, in which case s is in the range 2.5–3.3 [11]. In the case of Carbopol gels, various values for the exponent have been reported at different concentrations, in the range 0.87≤s≤2 (see [22] and references therein). Setting s=1 in Equation (2) yields

(3) $$u_{w}^{*}=\{\begin{array}{ll} 0, & \tau_{w}^{*}\leq \tau_{c}^{*}\\ \frac{\tau_{w}^{*}-\tau_{c}^{*}}{\beta^{*}}, & \tau_{w}^{*}>\tau_{c}^{*} \end{array},$$

which has been proposed for Newtonian fluids by Spikes and Granick [12], who tested the applicability of the above equation on experimental data and discussed possible physical mechanisms. When s=1 and $\tau_{c}^{*}=0$ the classical Navier slip [23] condition is recovered:

(4) $$u_{w}^{*}=\frac{\tau_{w}^{*}}{\beta^{*}}.$$

In this case, the slip coefficient is related to the slip or extrapolation length, $b^{*}$, defined as the characteristic length equal to the distance that the velocity profile at the wall must be extrapolated to reach zero, i.e., $b^{*}\equiv \eta^{*}/\beta^{*}$, and $\eta^{*}$ being the viscosity [11]. 

The two-branch form of the slip Equation (2) leads to some interesting theoretical as well as numerical difficulties, similar to those encountered with the discontinuous Bingham model. Different flow regimes are defined by critical values for the occurrence of slip along a wall. Moreover, in 2D and 3D problems, slip may occur only along unknown parts of the wall which is of interest from both the physics and the numerical points of view. Recently, analytical solutions of pressure-driven Newtonian flows in various geometries with wall slip governed by Equation (3) have been derived both for steady-state [24,25] and time-dependent [26] flows. Damianou et al. [16] solved the cessation of axisymmetric Newtonian Poiseuille flow and showed that if the initial pressure gradient is greater than the critical value for the occurrence of slip, then slip occurs only initially till a finite critical time at which slip ceases and cessation continues without slip. They also employed a regularized version of Equation (2) in order to numerically solve steady-state and time-dependent Poiseuille flows of a Herschel-Bulkley fluid [16]. 

Returning to viscoplastic materials exhibiting slip with non-zero slip yield stress, the relative value of $\tau_{c}^{*}$ with respect to the yield stress $\tau_{0}^{*}$ is of interest, since different flow situations may arise. In most experimental studies on various materials $\tau_{c}^{*}$ appears to be much lower than $\tau_{0}^{*}$ [20,21,27,28]. For example, for microgel pastes Seth et al. [29] reported values of $\tau_{c}^{*}$ and $\tau_{0}^{*}$ in the ranges 3.2–6 Pa and 53–117 Pa, respectively. Similarly, Piau [21] reported values in the ranges 0.23–23 Pa and 22–94 Pa. Daneshi et al. [22] reported that for water-based Carbopol gels with a concentration greater than 0.075%, the slip yield stress increases linearly with the yield stress, $\tau_{c}^{*}=(0.18\pm 0.02)\;\tau_{0}^{*}$, and roughly linearly with the solvent viscosity. 

Kalyon [13] states that wall slip is inevitable during the flow of viscoplastic fluids under stress magnitudes smaller than their yield stress values. Malkin and Patlazhan [11] note that wall slip with viscoplastic media takes place at low stresses and can exist in two different physical states below (solid-like) and above (liquid-like) the yield stress. In certain systems, such as microgels [18,29] and foams [30], slip appears to be significant for stresses below $\tau_{0}^{*}$ and to decrease and even disappear at high enough shear stresses. Hence, three slip regimes are identified with these systems: an initial sliding regime (no deformation) for $\tau^{*}<\tau_{0}^{*}$, an intermediate regime above $\tau_{0}^{*}$ where deformation is observed along with slip, and a regime where slip is suppressed and/or becomes negligible [7]. Similarly, Poumaere et al. [31] reported that wall slip effects in Carbopol gel flows are more pronounced in a range of low shear rates where the solid-fluid transition takes place and less important far above the yield point. 

Experimental methods for the measurement of the rheological properties of viscoplastic fluids have been recently reviewed by Ovarlez [32]. Circular Couette rheometers are very often employed for the rheological characterisation of yield-stress materials, in particular of drilling fluids and well cements [33,34,35]. The fluid sample is put in the gap between two coaxial cylinders one of which is rotating at constant angular velocity. The analysis of rheometric data becomes more complicated when slip occurs [7]. The presence of wall slip in rotational rheometers, evidenced by a reduction of the measured torque (shear stress) at a given shear rate, complicates the determination of the yield stress of viscoplastic materials [13] and prevents the accurate assessment of the solid-fluid transition [31]. For this reason, slip is often suppressed by adding roughness to the cylinder walls, mostly by means of sandpaper or ribs [36]. 

The effects of slip in circular Couette flow have been investigated by various groups. Yoshimura and Prud’homme [37] extended Mooney’s method to both Couette and parallel disk viscometers and presented an analysis of the Couette geometry that requires only two measurements rather than the three used by Mooney in order to assess wall slip of general materials. Yilmazer and Kalyon [17] generalized the above method. Their approach was later used by Bertola et al. [30] to measure the wall slip exhibited by pasty materials. Yeow et al. [38] proposed a procedure based on Tikhonov regularization to analyze Couette viscometry data in the presence of slip and extract the rheological property functions. They indicated that their method can be used to obtain estimates for the yield stress and the wall shear stress at the onset of slip. 

Hron et al. [39] derived analytical solutions of the Couette flow of Newtonian, power-law and second-grade fluids in the case of Navier slip on the boundaries. Ren and Zhu [40] derived solutions for an electrorheological fluid with Navier slip assuming that the yield stress is a function of the radial distance (i.e., $\tau_{0}^{*}=a^{*}/r^{*b}$). Philippou et al. [41] solved analytically both the steady-state and time-dependent Couette flows of a Newtonian fluid with wall slip following Equation (3), i.e., with non-zero slip yield stress, showing the existence of three steady-state regimes, defined by the critical values of the angular velocity at which slip starts at the two cylinders. It has been shown that during cessation, slip ceases first at the outer and then at the inner cylinder. 

The objective of the present work is to investigate the effect of wall slip with nonzero slip yield stress on the circular Couette flow of an ideal Bingham plastic and to identify the various flow regimes that arise depending on the relative values $\tau_{0}^{*}$ and $\tau_{c}^{*}$. Besides rheometry, this flow is encountered in industrial applications, such as electrorheological clutches [42], catalytic chemical reactors, filtration devices, liquid-liquid extractors, journal bearings, and oil and gas drilling [43]. 

In Section 2, the Couette flow with the inner cylinder rotating is solved in the presence of Navier slip (zero slip yield stress and s=1), in order to derive the basic solutions for comparison purposes. The case where the slip yield stress is non-zero and s=1 is studied in Section 3. The solutions when $\tau_{c}^{*}=\tau_{0}^{*}$ and $\tau_{c}^{*}<\tau_{0}^{*}$ are derived and the different flow regimes are discussed. 

## 2. Navier Slip 

**Figure 1:**
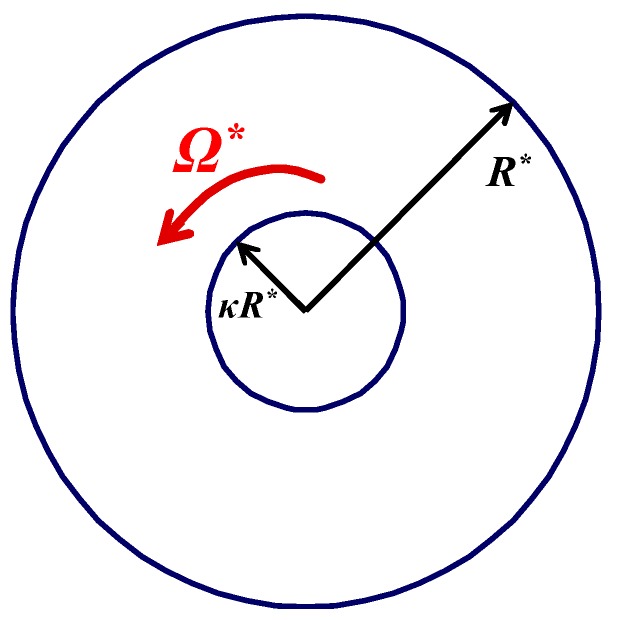
Geometry of circular Couette flow with the inner cylinder rotating.

We consider the steady-state flow of a Bingham plastic between two infinitely long co-axial cylinders of radii $\kappa R^{*}$ and $R^{*}$, where 0<κ<1. Since this is more common in rheometry [32], we examine here the case where the inner cylinder is rotating at a constant angular velocity $\Omega^{*}$ while the outer cylinder is fixed, as illustrated in Figure 1. Due to axisymmetry, the flow is one-dimensional with $u_{\theta }^{*}=u_{\theta }^{*}\left(r^{*}\right)$ and the *θ*-momentum equation yields

(5) $$\tau_{r\theta }^{*}=-\frac{c^{*}}{r^{*2}},$$

where $c^{*}$ is a positive constant to be determined from the boundary conditions. It is clear that the shear stress attains its maximum at the inner cylinder and decreases monotonically towards the outer cylinder. 

Let $\tau_{w1}^{*}$ and $\tau_{w2}^{*}$ denote the wall shear stresses along the inner and the outer cylinder, respectively. Hence,

(6) $$\tau_{w1}^{*}={\left|\tau_{r\theta }^{*}\right|}_{r^{*}=\kappa R^{*}}=\frac{c^{*}}{\kappa^{2}R^{*2}},\;\tau_{w2}^{*}={\left|\tau_{r\theta }^{*}\right|}_{r^{*}=R^{*}}=\frac{c^{*}}{R^{*2}}$$

and thus

(7) $$\tau_{w2}^{*}=\kappa^{2}\tau_{w1}^{*}.$$

We consider the general case where Navier slip occurs along both the inner and outer cylinders and denote the two slip velocities by $u_{w1}^{*}$ and $u_{w2}^{*}$, respectively. We allow the possibility of different slip coefficients along the two walls so that

(8) $$u_{wi}^{*}=\frac{\tau_{wi}^{*}}{\beta_{i}^{*}},\;\;i=1,2.$$

The conditions for the velocity at the two cylinders are as follows:

(9) $$u_{\theta }^{*}\left(\kappa R^{*}\right)=\Omega^{*}\kappa R^{*}-u_{w1}^{*}$$

and

(10) $$u_{\theta }^{*}\left(R^{*}\right)=u_{w2}^{*}.$$

**Figure 2:**
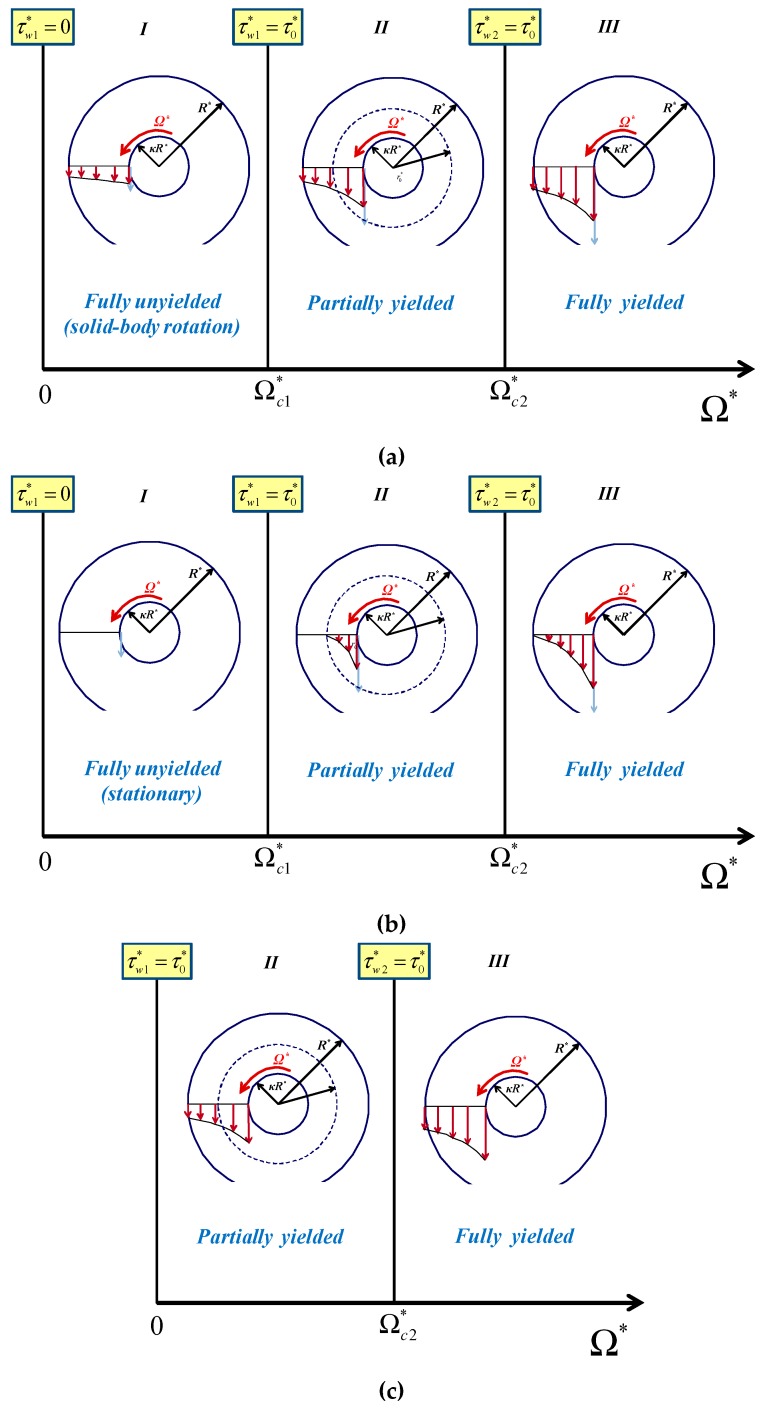
Flow regimes in the presence of Navier slip when the inner cylinder is rotating: (**a**) slip along both cylinders; the material rotates as a solid in Regime I and in the unyielded region of Regime II; (**b**) slip only along the inner cylinder; the material is stationary in Regime I and in the unyielded region of Regime II; (**c**) slip only along the outer cylinder; $\Omega_{c1}^{*}=0$ and Regime I is not observed. It should be noted that $\tau_{w2}^{*}=\tau_{0}^{*}$ is equivalent to $\tau_{w1}^{*}=\tau_{0}^{*}/\kappa^{2}$.

As illustrated in Figure 2a, three flow regimes are encountered as the wall shear stress increases:

(a) Regime I. When $\tau_{w1}^{*}\leq \tau_{0}^{*}$ all the material is unyielded rotating as a solid body with an angular velocity ${\Omega^{\prime }}^{*}$, which is smaller than the angular velocity of the inner cylinder (${\Omega^{\prime }}^{*}<\Omega^{*}$). In the case of no-slip along the outer cylinder, the material is stationary (Figure 2b), while in the case of no slip along both walls this regime is not observed (Figure 2c). It should be noted that the velocities sketched in Figure 2 are indicative sketches (i.e., not accurate); for example, in the regions of solid-body rotation the velocity is actually increasing with *r*.
(b) Regime II. When $\tau_{w1}^{*}>\tau_{0}^{*}$ and $\tau_{w2}^{*}\leq \tau_{0}^{*}$ the material in the gap is partially yielded, i.e., it yields only for $kR^{*}\leq r^{*}\leq r_{0}^{*}$, where $r_{0}^{*}$ is the outer radius of the yielded core, i.e., the radius at which $\tau^{*}=\left|\tau_{r\theta }^{*}\right|=\tau_{0}^{*}$. The material in the unyielded annulus $r_{0}^{*}\leq r^{*}\leq R^{*}$ rotates as a solid body when slip is imposed on the outer cylinder. Otherwise, the unyielded material is stationary (Figure 2b). As the inner wall shear stress is increased in this regime the radius $r_{0}^{*}$ increases from $\kappa R^{*}$ to $R^{*}$.
(c) Regime III. When $\tau_{w2}^{*}\geq \tau_{0}^{*}$ the material in the gap is fully yielded.

As depicted in Figure 2, the three regimes are defined by the two critical values of the angular velocity, $\Omega_{c1}^{*}$ and $\Omega_{c2}^{*}$, which correspond to $\tau_{w1}^{*}=\tau_{0}^{*}$ and $\tau_{w2}^{*}=\tau_{0}^{*}$, respectively (the latter condition is equivalent to $\tau_{w1}^{*}=\tau_{0}^{*}/\kappa^{2}$).

In Regime I, the material rotates as solid body so that

(11) $$u_{\theta }^{*}\left(r^{*}\right)={\Omega^{\prime }}^{*}r^{*}.$$

Applying Conditions (9) and (10), one gets

(12) $${\Omega^{\prime }}^{*}\kappa R^{*}=\Omega^{*}\kappa R^{*}-u_{w1}^{*}$$

and

(13) $${\Omega^{\prime }}^{*}R^{*}=u_{w2}^{*}.$$

It is clear that if there is no slip along the outer cylinder ($\beta_{2}^{*}\to \infty$) the angular velocity ${\Omega^{\prime }}^{*}$ vanishes and the material is stationary, despite the fact that the inner cylinder is rotating. Since ${\Omega^{\prime }}^{*}=0$, Equation (12) gives

(14) $$u_{w1}^{*}=\frac{\tau_{w1}^{*}}{\beta_{1}^{*}}=\Omega^{*}\kappa R^{*},$$

which simply says that the fluid slips fully to remain stagnant despite the rotation of the inner cylinder (Figure 2b). For the first critical angular velocity (which corresponds to $\tau_{w1}^{*}=\tau_{0}^{*}$) one finds

(15) $$\Omega_{c1}^{*}=\frac{\tau_{0}^{*}}{\beta_{1}^{*}\kappa R^{*}}.$$

If, however, slip is allowed at the outer cylinder, Equation (13) gives

(16) $${\Omega^{\prime }}^{*}R^{*}=u_{w2}^{*}=\frac{\tau_{w2}^{*}}{\beta_{2}^{*}}=\frac{\kappa^{2}\tau_{w1}^{*}}{\beta_{2}^{*}}=\frac{\kappa^{2}\beta_{1}^{*}u_{w1}^{*}}{\beta_{2}^{*}}\;\Rightarrow u_{w1}^{*}=\frac{\beta_{2}^{*}{\Omega^{\prime }}^{*}R^{*}}{\kappa^{2}\beta_{1}^{*}}.$$

Substituting into Equation (12) one finds

(17) $${\Omega^{\prime }}^{*}=\frac{\Omega^{*}}{1+\frac{\beta_{2}^{*}}{\kappa^{3}\beta_{1}^{*}}}.$$

Therefore, in Regime I, the velocity is given by:

(18) $$u_{\theta }^{*}(r^{*})=\frac{\Omega^{*}r^{*}}{1+\frac{\beta_{2}^{*}}{\kappa^{3}\beta_{1}^{*}}}.$$

In the case of no slip along the inner cylinder ($\beta_{1}^{*}\to \infty$), ${\Omega^{\prime }}^{*}=\Omega^{*}$ and $u_{\theta }^{*}(r^{*})=\Omega^{*}r^{*}$ (solid-body rotation). For the slip velocities one gets:

(19) $$u_{w1}^{*}=\frac{\Omega^{*}\kappa R^{*}}{1+\frac{\kappa^{3}\beta_{1}^{*}}{\beta_{2}^{*}}},\;u_{w2}^{*}=\frac{\Omega^{*}R^{*}}{1+\frac{\beta_{2}^{*}}{\kappa^{3}\beta_{1}^{*}}}.$$

Setting $\tau_{w1}^{*}=\tau_{0}^{*}=\beta_{1}^{*}u_{w1}^{*}$ gives the first critical angular velocity

(20) $$\Omega_{c1}^{*}=\frac{\tau_{0}^{*}}{\beta_{1}^{*}\kappa R^{*}}\left(1+\frac{\kappa^{3}\beta_{1}^{*}}{\beta_{2}^{*}}\right).$$

Let us now examine what happens in Regime II where $\Omega^{*}\geq \Omega_{c1}^{*}$. In the yielded region ($\kappa R^{*}\leq r^{*}\leq r_{0}^{*}$), the shear stress component of the stress tensor in Equation (1) becomes

(21) $$\tau_{r\theta }^{*}=\left(\frac{\tau_{0}^{*}}{{\dot{\gamma }}^{*}}+\mu^{*}\right)r^{*}\frac{d}{dr}\left(\frac{u_{\theta }^{*}}{r^{*}}\right).$$

Given that the inner cylinder is rotating and the outer one is fixed, the angular velocity $u_{\theta }^{*}/r^{*}$ is a decreasing function of $r^{*}$ and thus

(22) $${\dot{\gamma }}^{*}=r^{*}\left|\frac{d}{dr^{*}}\left(\frac{u_{\theta }^{*}}{r^{*}}\right)\right|=-r^{*}\frac{d}{dr^{*}}\left(\frac{u_{\theta }^{*}}{r^{*}}\right)$$

and

(23) $$\tau_{r\theta }^{*}=-\tau_{0}^{*}+\mu^{*}r^{*}\frac{d}{dr^{*}}\left(\frac{u_{\theta }^{*}}{r^{*}}\right).$$

From Equations (5) and (23) we have

(24) $$\frac{d}{dr^{*}}\left(\frac{u_{\theta }^{*}}{r^{*}}\right)=\frac{1}{\mu^{*}}\left(\frac{\tau_{0}^{*}}{r^{*}}-\frac{c^{*}}{r^{*3}}\right),$$

which upon integration yields

(25) $$u_{\theta }^{*}(r^{*})=\frac{1}{\mu^{*}}\left(\tau_{0}^{*}r^{*}\ln r^{*}+\frac{c^{*}}{2r^{*}}\right)+c_{1}^{*}r^{*},$$

where $c_{1}^{*}$ is the integration constant.

Applying the boundary Condition (12) at the inner cylinder ($r^{*}=\kappa R^{*}$) and using

(26) $$u_{w1}^{*}=\frac{\tau_{w1}^{*}}{\beta_{1}^{*}}=\frac{c^{*}}{\beta_{1}^{*}\kappa^{2}R^{*2}}$$

we get from Equation (25):

(27) $$c_{1}^{*}=\Omega -\frac{1}{\mu^{*}}\tau_{0}^{*}\ln\left(\kappa R^{*}\right)-\frac{c^{*}(1+2B_{1})}{2\mu^{*}\kappa^{2}R^{*2}},$$

where $B_{1}$ is the dimensionless inner slip number defined by

(28) $$B_{1}\equiv \frac{\mu^{*}}{\beta_{1}^{*}\kappa R^{*}}.$$

Substituting Equation (27) into Equation (25) results in the following expression for the velocity in the yielded region ($\kappa R^{*}\leq r^{*}\leq r_{0}^{*}$):

(29) $$u_{\theta }^{*}(r^{*})=r^{*}\left[\Omega^{*}+\frac{\tau_{0}^{*}}{\mu^{*}}\ln\left(\frac{r^{*}}{\kappa R^{*}}\right)-\frac{c^{*}}{2\mu^{*}\kappa^{2}R^{*2}}\left(1+2B_{1}-\frac{\kappa^{2}R^{*2}}{r^{*2}}\right)\right].$$

From Equation (5) it is deduced that

(30) $$c^{*}=\tau_{0}^{*}r_{0}^{*2}$$

and Equation (29) thus becomes

(31) $$u_{\theta }^{*}(r^{*})=\frac{\tau_{0}^{*}}{\mu^{*}}r^{*}\left[\frac{\Omega^{*}\mu^{*}}{\tau_{0}^{*}}+\ln\left(\frac{r^{*}}{\kappa R^{*}}\right)-\frac{r_{0}^{*2}}{2\kappa^{2}R^{*2}}\left(1+2B_{1}-\frac{\kappa^{2}R^{*2}}{r^{*2}}\right)\right].$$

In the unyielded region ($r_{0}^{*}\leq r^{*}\leq R^{*}$), the material rotates as a solid with angular velocity ${\Omega^{\prime }}^{*}$. The continuity of the velocity requires that $u_{\theta }^{*}(r_{0}^{*})={\Omega^{\prime }}^{*}r_{0}^{*}$ and therefore

(32) $${\Omega^{\prime }}^{*}=\Omega^{*}+\frac{\tau_{0}^{*}}{\mu^{*}}\left[\ln\left(\frac{r_{0}^{*}}{\kappa R^{*}}\right)-(1+2B_{1})\frac{r_{0}^{*2}}{2\kappa^{2}R^{*2}}+\frac{1}{2}\right].$$

Applying the boundary Condition (13) at the outer cylinder along with the Navier slip condition we get

(33) $${\Omega^{\prime }}^{*}=\frac{\tau_{0}^{*}r_{0}^{*2}}{\beta_{2}^{*}R^{*3}}.$$

Combining Equations (32) and (33) leads to the following nonlinear equation

(34) $$\frac{\Omega^{*}\mu^{*}}{\tau_{0}^{*}}+\ln\left(\frac{r_{0}^{*}}{\kappa R^{*}}\right)-(1+2B_{1}+2\kappa^{3}B_{2})\frac{r_{0}^{*2}}{2\kappa^{2}R^{*2}}+\frac{1}{2}=0$$

for the yield radius $r_{0}^{*}$, where $B_{2}$ is the dimensionless outer slip number

(35) $$B_{2}\equiv \frac{\mu^{*}}{\beta_{2}^{*}\kappa R^{*}}.$$

Hence, the velocity in Regime II is:

(36) $$u_{\theta }^{*}(r^{*})=\frac{\tau_{0}^{*}}{\mu^{*}}r^{*}\{\begin{array}{cc} \frac{\Omega^{*}\mu^{*}}{\tau_{0}^{*}}+\ln\left(\frac{r^{*}}{\kappa R^{*}}\right)-\frac{r_{0}^{*2}}{2\kappa^{2}R^{*2}}\left(1+2B_{1}-\frac{\kappa^{2}R^{*2}}{r^{*2}}\right), & \kappa R^{*}\leq r^{*}\leq r_{0}^{*}\\ \kappa B_{2}\frac{r_{0}^{*2}}{R^{*2}}, & r_{0}^{*}<r^{*}\leq R^{*} \end{array}.$$

The slip velocities in this regime are given by

(37) $$u_{w1}^{*}=\frac{\tau_{0}^{*}r_{0}^{*2}}{\beta_{1}^{*}\kappa^{2}R^{*2}},\;u_{w2}^{*}=\frac{\tau_{0}^{*}r_{0}^{*2}}{\beta_{2}^{*}R^{*2}}.$$

The second critical angular velocity $\Omega_{c2}^{*}$ can be obtained by setting $r_{0}^{*}=R^{*}$ in Equation (34) (which is equivalent to $\tau_{w2}^{*}=\tau_{0}^{*}$):

(38) $$\Omega_{c2}^{*}=\frac{\tau_{0}^{*}}{\mu^{*}}\left[\frac{1}{2}\left(\frac{1+2B_{1}+2\kappa^{3}B_{2}}{\kappa^{2}}-1\right)-\ln\left(\frac{1}{\kappa }\right)\right].$$

In Regime III ($\Omega^{*}>\Omega_{c2}^{*}$), the boundary condition at the fixed outer cylinder is

(39) $$u_{\theta }^{*}\left(R^{*}\right)=u_{w2}^{*},$$

where

(40) $$u_{w2}^{*}=\frac{\tau_{w2}^{*}}{\beta_{2}^{*}}=\frac{c^{*}}{\beta_{2}^{*}R^{*2}}.$$

Substituting into Equation (29) gives

(41) $$c^{*}=\frac{2\mu^{*}\kappa^{2}R^{*2}}{1-\kappa^{2}+2B_{1}+2B_{2}\kappa^{3}}\left(\Omega^{*}+\frac{\tau_{0}^{*}}{\mu^{*}}\ln\frac{1}{\kappa }\right)$$

Inserting Equation (41) into Equation (29) we get the velocity distribution in the fully-yielded regime:

(42) $$u_{\theta }^{*}(r^{*})=\frac{\tau_{0}^{*}}{\mu^{*}}r^{*}\left[\frac{\Omega^{*}\mu^{*}}{\tau_{0}^{*}}+\ln\frac{r^{*}}{\kappa R^{*}}-\frac{\frac{\Omega^{*}\mu^{*}}{\tau_{0}^{*}}+\ln\frac{1}{\kappa }}{1-\kappa^{2}+2B_{1}+2B_{2}\kappa^{3}}\left(1+2B_{1}-\frac{\kappa^{2}R^{*2}}{r^{*2}}\right)\right].$$

For the two slip velocities we now have:

(43) $$u_{w1}^{*}=\frac{2R^{*}\tau_{0}^{*}\kappa B_{1}}{\mu^{*}(1-\kappa^{2}+2B_{1}+2\kappa^{3}B_{2})}\left(\frac{\Omega^{*}\mu^{*}}{\tau_{0}^{*}}+\ln\frac{1}{\kappa }\right),\;u_{w2}^{*}=\frac{2R^{*}\tau_{0}^{*}\kappa^{3}B_{2}}{\mu^{*}(1-\kappa^{2}+2B_{1}+2\kappa^{3}B_{2})}\left(\frac{\Omega^{*}\mu^{*}}{\tau_{0}^{*}}+\ln\frac{1}{\kappa }\right)$$

Let us summarize the solution in its dimensionless form by scaling $r^{*}$ by $R^{*}$, $u_{\theta }^{*}$ by $\tau_{0}^{*}R^{*}/\mu^{*}$, $\Omega^{*}$ by $\tau_{0}^{*}/\mu^{*}$ and $\tau_{r\theta }^{*}$ by $\tau_{0}^{*}$. The dimensionless critical angular velocities are then given by:

(44) $$\Omega_{c1}=B_{1}+\kappa^{3}B_{2}$$

and

(45) $$\Omega_{c2}=\frac{1}{2\kappa^{2}}(1+2B_{1}+2\kappa^{3}B_{2})-\ln\frac{1}{\kappa }-\frac{1}{2}.$$

In Regime I ($\Omega \leq \Omega_{c1}$),

(46) $$u_{\theta }(r)=\frac{\Omega \;r}{1+\frac{B_{1}}{\kappa^{3}B_{2}}}$$

and

(47) $$u_{w1}=\frac{\Omega \kappa }{1+\frac{\kappa^{3}B_{2}}{B_{1}}},\;u_{w2}=\frac{\Omega }{1+\frac{B_{1}}{\kappa^{3}B_{2}}}.$$

In Regime II ($\Omega_{c1}<\Omega \leq \Omega_{c2}$),

(48) $$u_{\theta }(r)=r\{\begin{array}{cc} \Omega +\ln\frac{r}{\kappa }-\frac{r_{0}^{2}}{2\kappa^{2}}\left(1+2B_{1}-\frac{\kappa^{2}}{r^{2}}\right), & \kappa \leq r\leq r_{0}\\ \kappa B_{2}r_{0}^{2}, & r_{0}<r\leq 1 \end{array},$$

where $r_{0}$ is the root of

(49) $$\Omega +\ln\frac{r_{0}}{\kappa }-(1+2B_{1}+2\kappa^{3}B_{2})\frac{r_{0}^{2}}{2\kappa^{2}}+\frac{1}{2}=0$$

and the two slip velocities are given by:

(50) $$u_{w1}=\frac{1}{\kappa }B_{1}r_{0}^{2},\;u_{w2}=\kappa B_{2}r_{0}^{2}.$$

Finally, in Regime III ($\Omega >\Omega_{c2}$)

(51) $$u_{\theta }(r)=r\left[\Omega +\ln\frac{r}{\kappa }-\frac{\Omega +\ln\frac{1}{\kappa }}{1-\kappa^{2}+2B_{1}+2B_{2}\kappa^{3}}\left(1+2B_{1}-\frac{\kappa^{2}}{r^{2}}\right)\right]$$

and

(52) $$u_{w1}=\frac{2\kappa B_{1}}{1-\kappa^{2}+2B_{1}+2\kappa^{3}B_{2}}\left(\Omega +\ln\frac{1}{\kappa }\right),\;u_{w2}=\frac{2\kappa^{3}B_{2}}{1-\kappa^{2}+2B_{1}+2\kappa^{3}B_{2}}\left(\Omega +\ln\frac{1}{\kappa }\right).$$

It is easily verified that when $\Omega =\Omega_{c1}$ the inner slip velocity is $u_{w1}=\kappa B_{1}$; when $\Omega =\Omega_{c2}$, $u_{w1}=B_{1}/\kappa$. In all cases, $u_{w2}=\kappa^{2}B_{2}u_{w1}/B_{1}$. By setting $B_{1}=B_{2}=0$, one obtains the classical no-slip solutions in Regimes II and III. 

**Figure 3:**
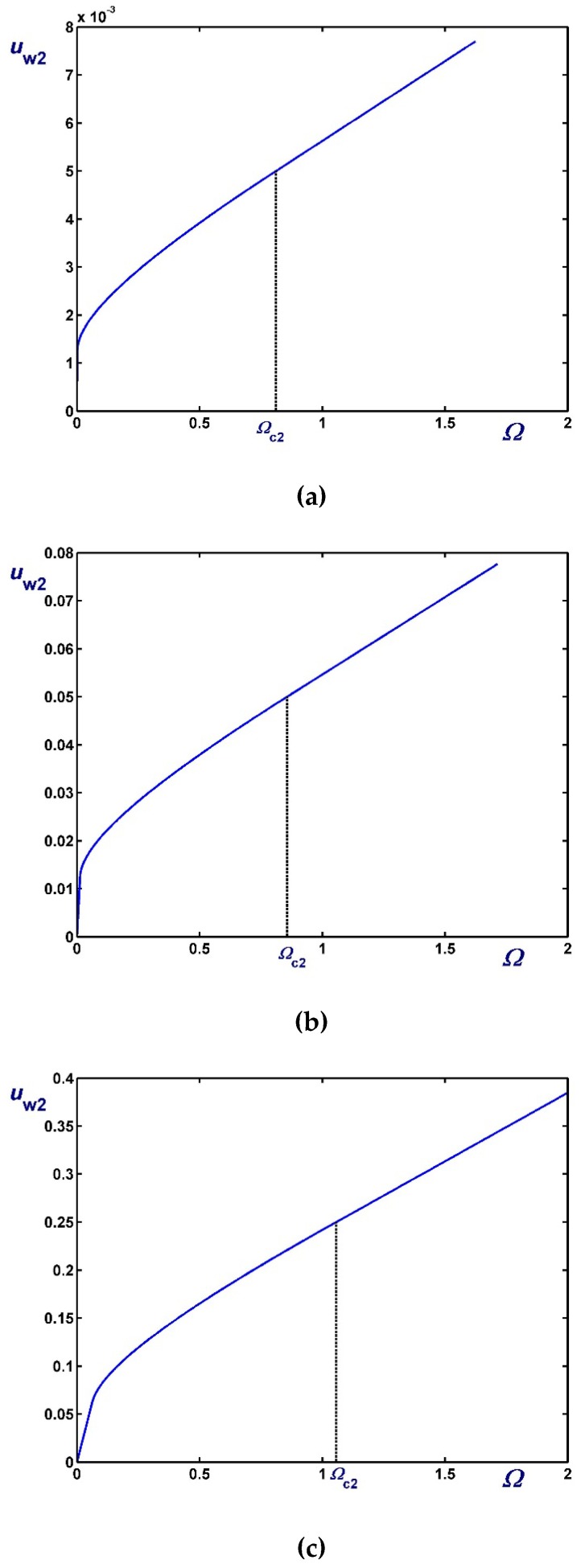
Outer wall slip velocity for κ=0.5,$B_{1}=0$ (no slip along the rotating inner cylinder) and Navier slip along the fixed outer cylinder: (**a**) $B_{2}=0.01$ (weak slip); (**b**) $B_{2}=0.1$ (moderate slip); (**c**) $B_{2}=0.5$ (strong slip).

**Figure 4:**
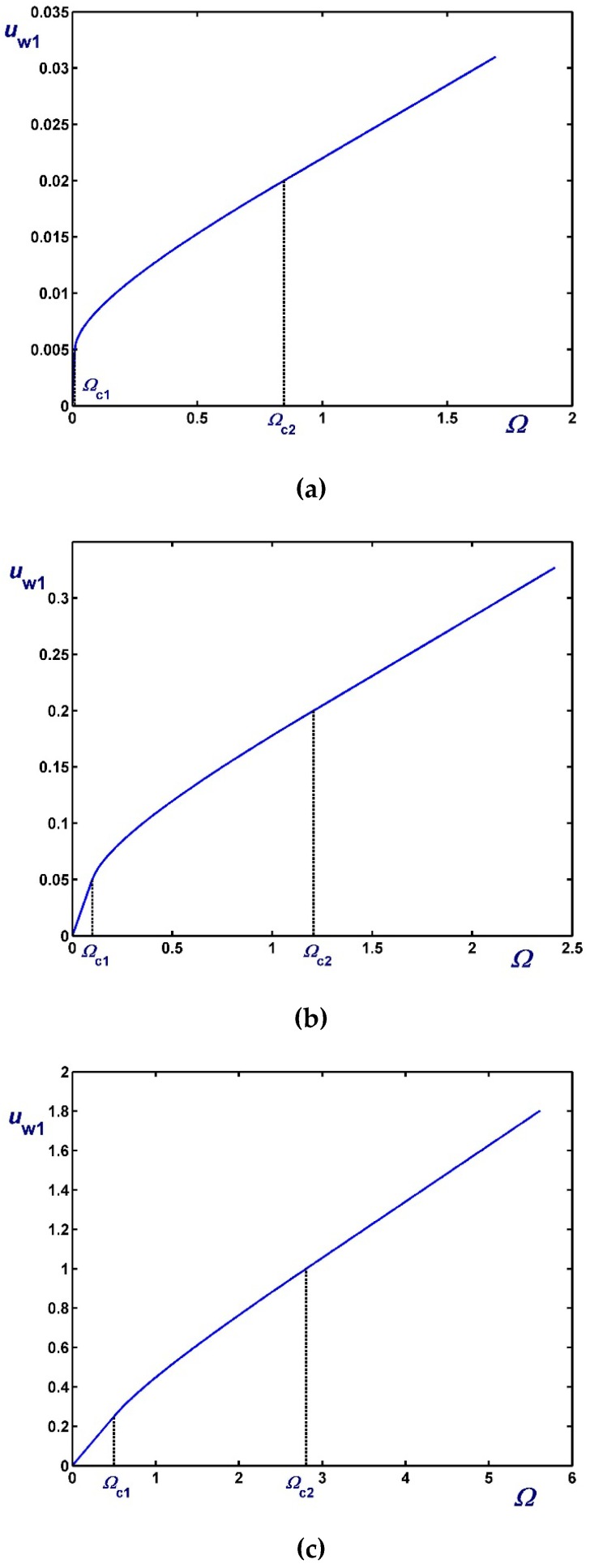
Inner wall slip velocity for κ=0.5, $B_{2}=0$ (no slip along the fixed outer cylinder), and Navier slip along the rotating inner cylinder: (**a**) $B_{1}=0.01$ (weak slip); (**b**) $B_{1}=0.1$ (moderate slip); (**c**) $B_{1}=0.5$ (strong slip).

**Figure 5:**
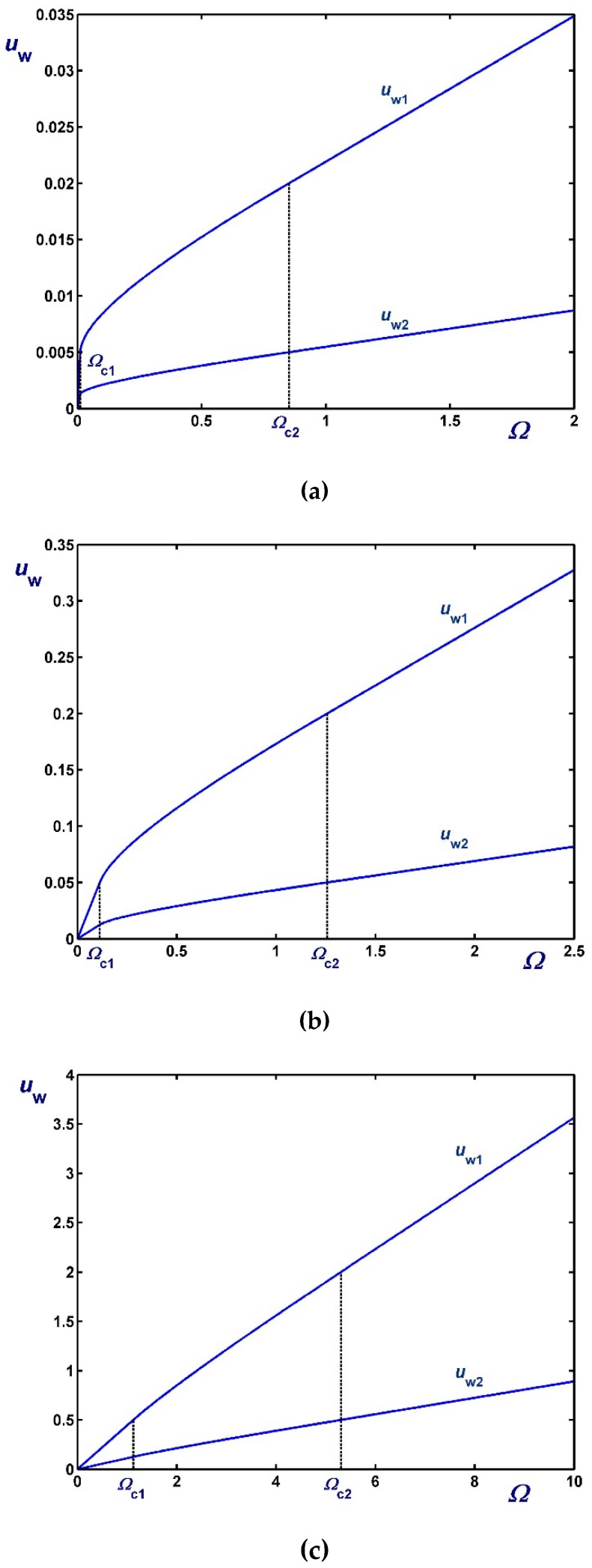
Slip velocities for κ=0.5 and Navier slip along both cylinders: (**a**) $B_{1}=B_{2}=0.01$ (weak slip); (**b**) $B_{1}=B_{2}=0.1$ (moderate slip); (**c**) $B_{1}=B_{2}=1$ (strong slip).

Figure 3, Figure 4 and Figure 5 show the effect of the dimensionless angular velocity Ω in a rheometer with κ=0.5. Three special cases are considered, i.e., no slip along the rotating cylinder ($B_{1}=0$) in Figure 3, no slip along the fixed cylinder ($B_{2}=0$) in Figure 4, and slip along both cylinders with equal slip coefficients ($B_{1}=B_{2}$) in Figure 5. One can observe that the (non-zero) slip velocity increases rapidly and eventually (in Regime III) varies linearly with Ω. When $B_{1}=0$ (Figure 3), Regime I is not observed ($\Omega_{c1}^{}=0$). 

**Figure 6:**
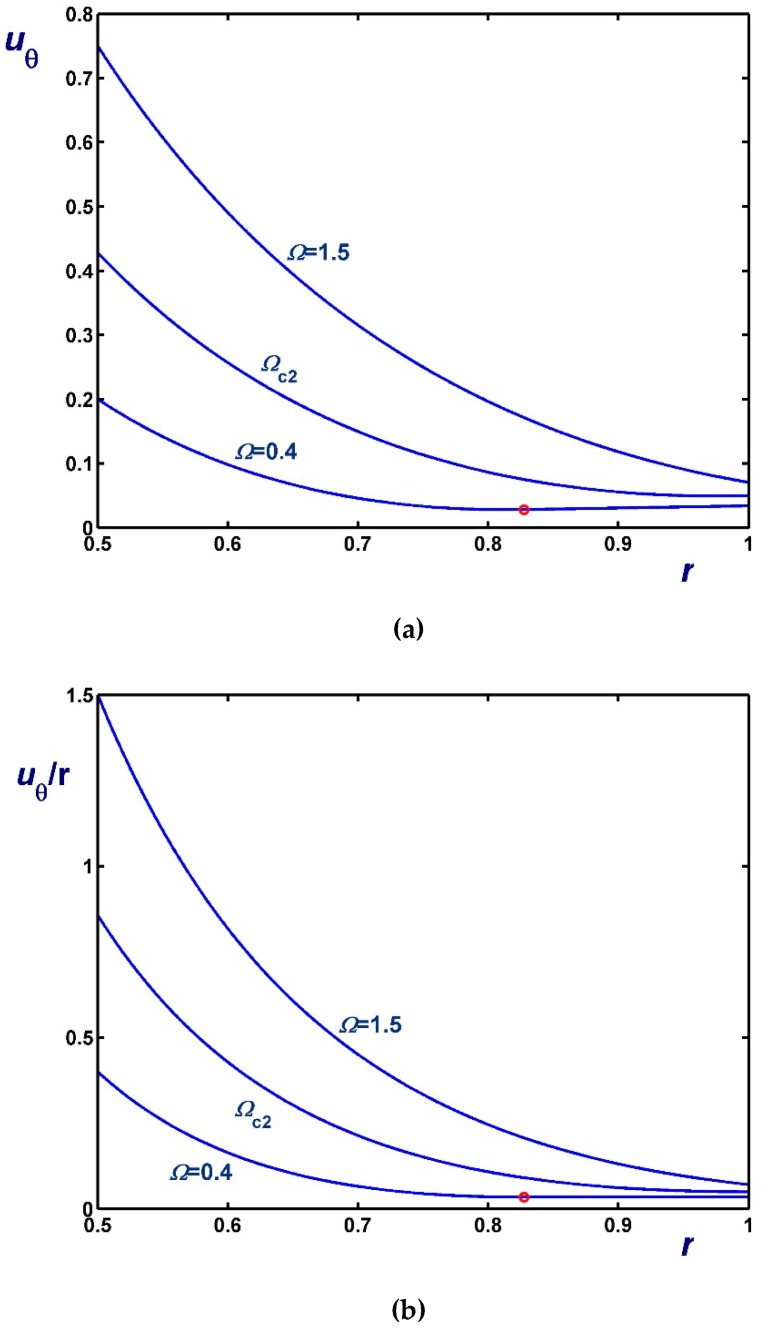
Velocity profiles in circular Couette flow of a Bingham plastic with Navier slip along the fixed outer cylinder with κ=0.5, $B_{1}=0$ and $B_{2}=0.1$ : (**a**) azimuthal velocity; (**b**) angular velocity; $\Omega_{c1}=0$ and $\Omega_{c2}=0.8569$. The red circle indicates the yield point for the velocity profile corresponding to Regime II (Regime I is not observed).

**Figure 7:**
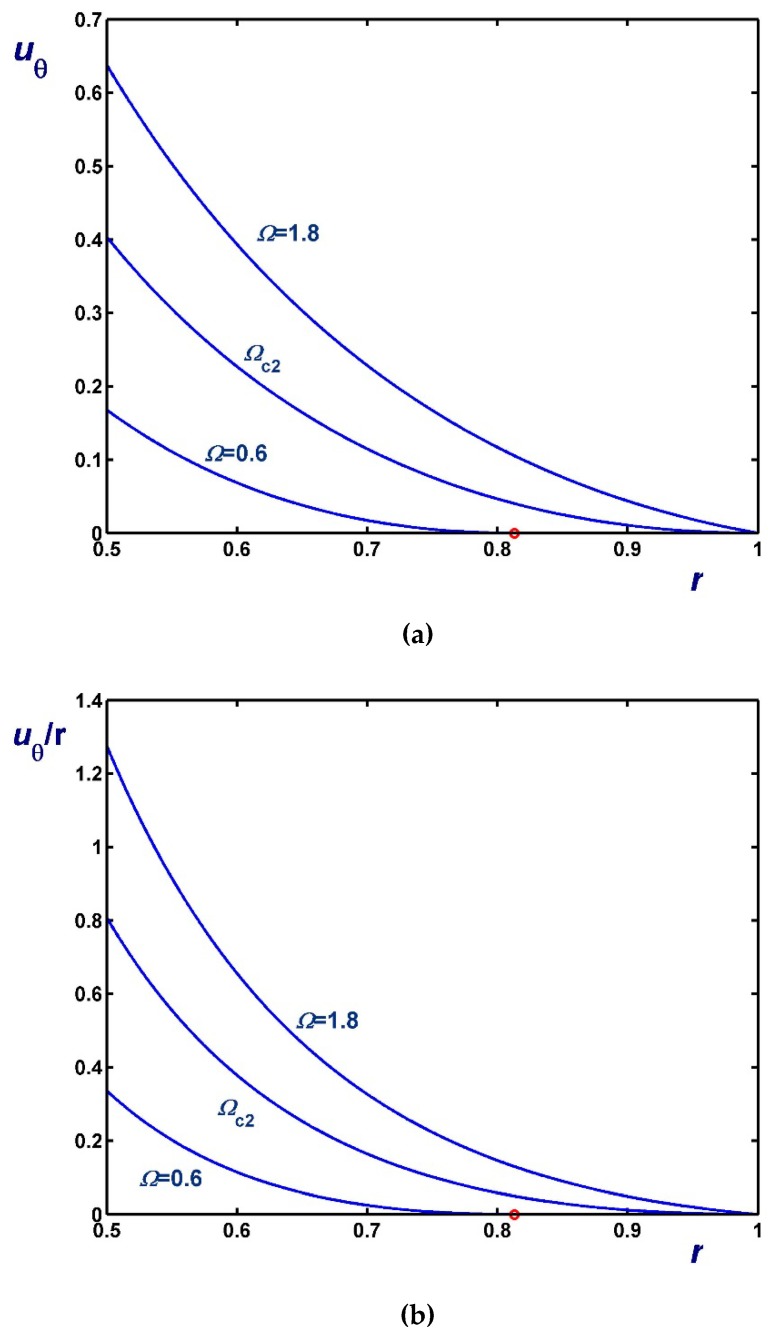
Velocity profiles in circular Couette flow of a Bingham plastic with Navier slip along the rotating inner cylinder with κ=0.5, $B_{1}=0.1$ and $B_{2}=0$ : (**a**) azimuthal velocity; (**b**) angular velocity; $\Omega_{c1}=0.1$ and $\Omega_{c2}=1.20685$. The red circle indicates the yield point for the velocity profile corresponding to Regime II (In Regime I the velocity of the fluid is zero).

**Figure 8:**
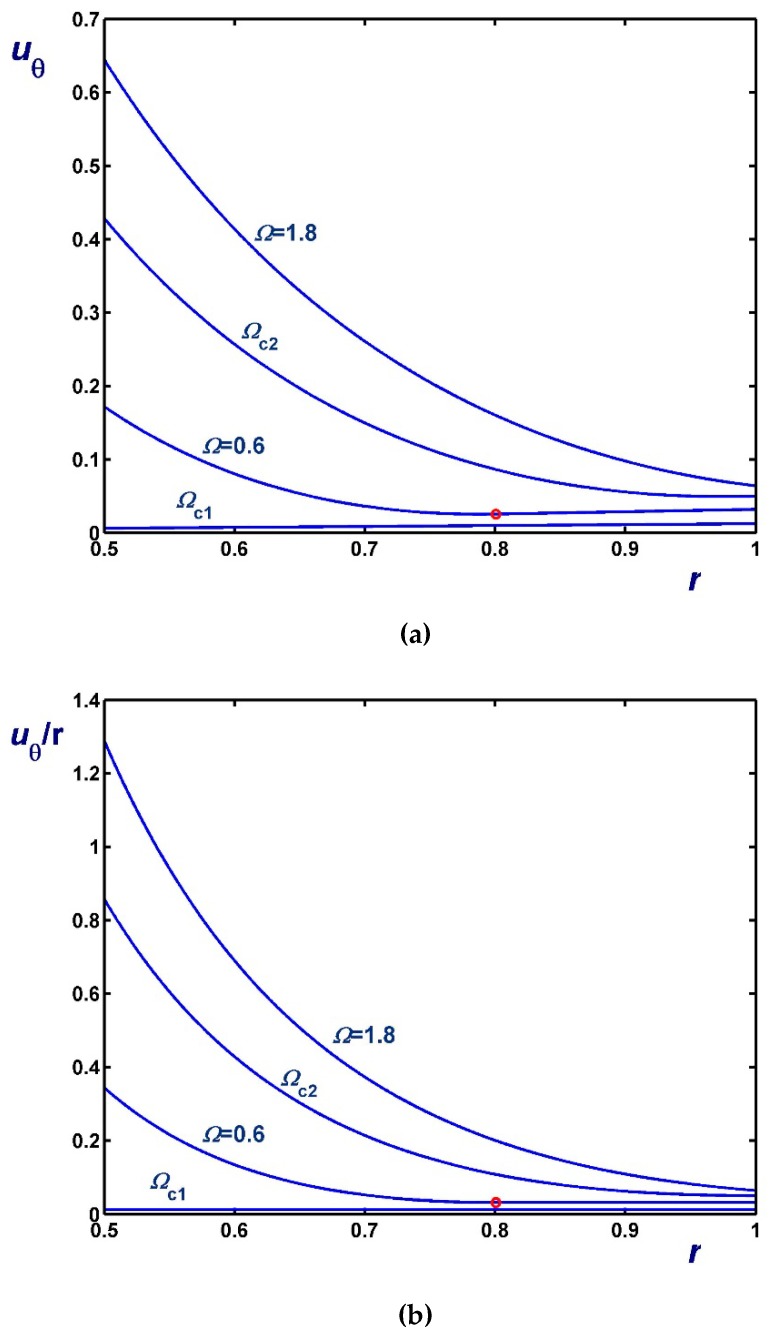
Velocity profiles in circular Couette flow of a Bingham plastic with Navier slip along both cylinders with κ=0.5 and $B_{1}=B_{2}=0.1$ : (**a**) azimuthal velocity; (**b**) angular velocity; $\Omega_{c1}=0.1125$ and $\Omega_{c2}=1.25685$. The red circle indicates the yield point for the velocity profile corresponding to Regime II; in Regime I, the fluid rotates as a solid body.

Figure 6, Figure 7 and Figure 8 illustrate the effect of Ω on the azimuthal ($u_{\theta }$) and angular ($u_{\theta }/r$) velocity profiles. Figure 6 shows results with $B_{1}=0$ (no-slip along the rotating cylinder) and $B_{2}=0.1$ for three values of Ω, including $\Omega_{c2}$. Note, in particular, that the velocity for Ω=0.4 (Regime II) increases with *r* in the unyielded region (solid-body rotation). Figure 7 shows results with $B_{2}=0$ (no-slip along the outer cylinder) and $B_{1}=0.1$ while Figure 8 shows results with $B_{1}=B_{2}=0.1$. In the latter figure, the profiles for $\Omega =\Omega_{c1}$ are shown; $u_{\theta }/r$ is finite and flat in this case, since the material is unyielded. 

## 3. Solution with Non-Zero Slip Yield Stress

The Navier-slip case analyzed in Section 2 is the special case of slip Equation (3) when the slip yield stress vanishes, $\tau_{c}^{*}=0$. Introducing a non-zero slip yield stress allows various possibilities depending on the relative values of $\tau_{0}^{*}$ and $\tau_{c}^{*}$. The case $\tau_{c}^{*}=\tau_{0}^{*}$, which is the simplest of all, since material yielding and wall slip occur simultaneously, is examined first. Then, the case $\tau_{c}^{*}<\tau_{0}^{*}$, which is more relevant to experimental observations [20,22], is analyzed. 

### 3.1. The Case $\tau_{c}^{*}=\tau_{0}^{*}$

We consider the general case where slip with non-zero slip yield stress occurs along both the inner and outer cylinders and allow the possibility of different slip coefficients along the two walls so that

(53) $$u_{wi}^{*}=\{\begin{array}{ll} 0, & \tau_{wi}^{*}\leq \tau_{0}^{*}\\ \frac{\tau_{wi}^{*}-\tau_{0}^{*}}{\beta_{i}^{*}}, & \tau_{wi}^{*}>\tau_{0}^{*} \end{array},\;i=1,2.$$

**Figure 9:**
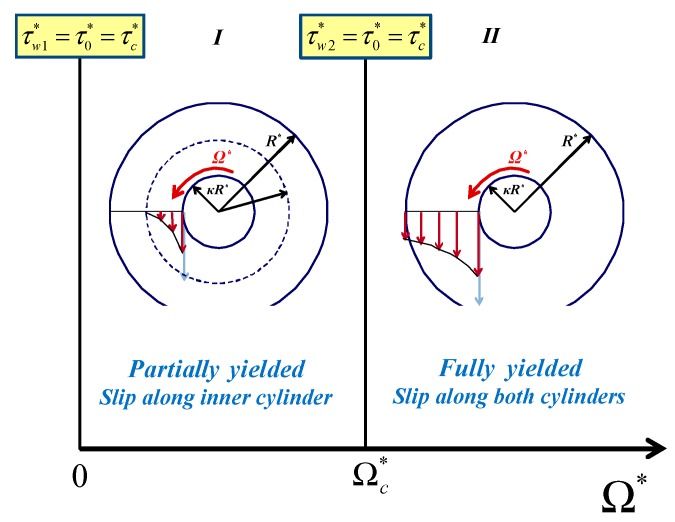
Flow regimes in circular Couette flow of a Bingham plastic in the presence of wall slip with non-zero slip yield stress, when $\tau_{c}^{*}=\tau_{0}^{*}$. The inner cylinder is rotating and the outer one is at rest.

Two flow regimes are encountered in this case, which are illustrated in Figure 9. In Regime I, the material is partially yielded and slip is observed only at the inner wall. Hence, in the unyielded region $r_{0}^{*}\leq r^{*}\leq R^{*}$ the material is stagnant. In Regime II, the material is fully-yielded and slip occurs along both walls. The critical angular velocity $\Omega_{c}^{*}$ defining the two regimes is the angular velocity at which $\tau_{w2}^{*}=\tau_{0}^{*}=\tau_{c}^{*}$. 

Since the derivation of the solution follows the same steps as in Section 2, it is omitted here and only the final dimensionless equations, with the same scales, are provided. It should be noted that in this general case $B_{1}>0$ and $B_{2}>0$. The critical angular velocity is given by

(54) $$\Omega_{c}=\frac{1}{2\kappa^{2}}\left(1+2B_{1}\right)(1-\kappa^{2})-\ln\frac{1}{\kappa }.$$

In the partially yielded Regime I, the velocity is given by

(55) $$u_{\theta }(r)=r\{\begin{array}{cc} \Omega +\ln\frac{r}{\kappa }+B_{1}-\frac{r_{0}^{2}}{2\kappa^{2}}\left(1+2B_{1}-\frac{\kappa^{2}}{r^{2}}\right), & \kappa \leq r\leq r_{0}\\ 0, & r_{0}<r\leq 1 \end{array},$$

where $r_{0}$ is the root of

(56) $$\Omega +\ln\frac{r_{0}}{\kappa }-\frac{1}{2\kappa^{2}}\left(1+2B_{1}\right)(r_{0}^{2}-\kappa^{2})=0.$$

Moreover,

(57) $$\tau_{w1}=\frac{r_{0}^{2}}{\kappa^{2}}$$

and

(58) $$u_{w1}=\frac{B_{1}(r_{0}^{2}-\kappa^{2})}{\kappa }.$$

In the fully-yielded Regime II, the velocity is given by

(59) $$u_{\theta }(r)=r\left[\Omega +\ln\frac{r}{\kappa }+B_{1}-\frac{\Omega +\ln\frac{1}{\kappa }+B_{1}+B_{2}\kappa }{1-\kappa^{2}+2B_{1}+2B_{2}\kappa^{3}}\left(1+2B_{1}-\frac{\kappa^{2}}{r^{2}}\right)\right],$$

(60) $$\tau_{w1}=\frac{2\left(\Omega +\ln\frac{1}{\kappa }+B_{1}+B_{2}\kappa \right)}{1-\kappa^{2}+2B_{1}+2B_{2}\kappa^{3}},\;\tau_{w2}=\kappa^{2}\tau_{w1},$$

(61) $$u_{w1}=\frac{\kappa B_{1}\left[2\left(\Omega +\ln\frac{1}{\kappa }\right)-(1-\kappa^{2})(1-2B_{2}\kappa )\right]}{1-\kappa^{2}+2B_{1}+2B_{2}\kappa^{3}}$$

and

(62) $$u_{w2}=\frac{\kappa B_{2}\left[2\kappa^{2}\left(\Omega +\ln\frac{1}{\kappa }\right)-(1-\kappa^{2})(1+2B_{1})\right]}{1-\kappa^{2}+2B_{1}+2B_{2}\kappa^{3}}.$$

The two slip velocities are now related as follows:

(63) $$u_{w2}=\kappa B_{2}\left(\frac{\kappa u_{w1}}{B_{1}}-1+\kappa^{2}\right).$$

The expressions corresponding to various special cases, such as slip along the inner cylinder only ($B_{2}=0$), slip along the outer cylinder only ($B_{1}=0$), equal slip coefficients along the two cylinders ($B_{1}=B_{2}=B$), are easily deduced. 

**Figure 10:**
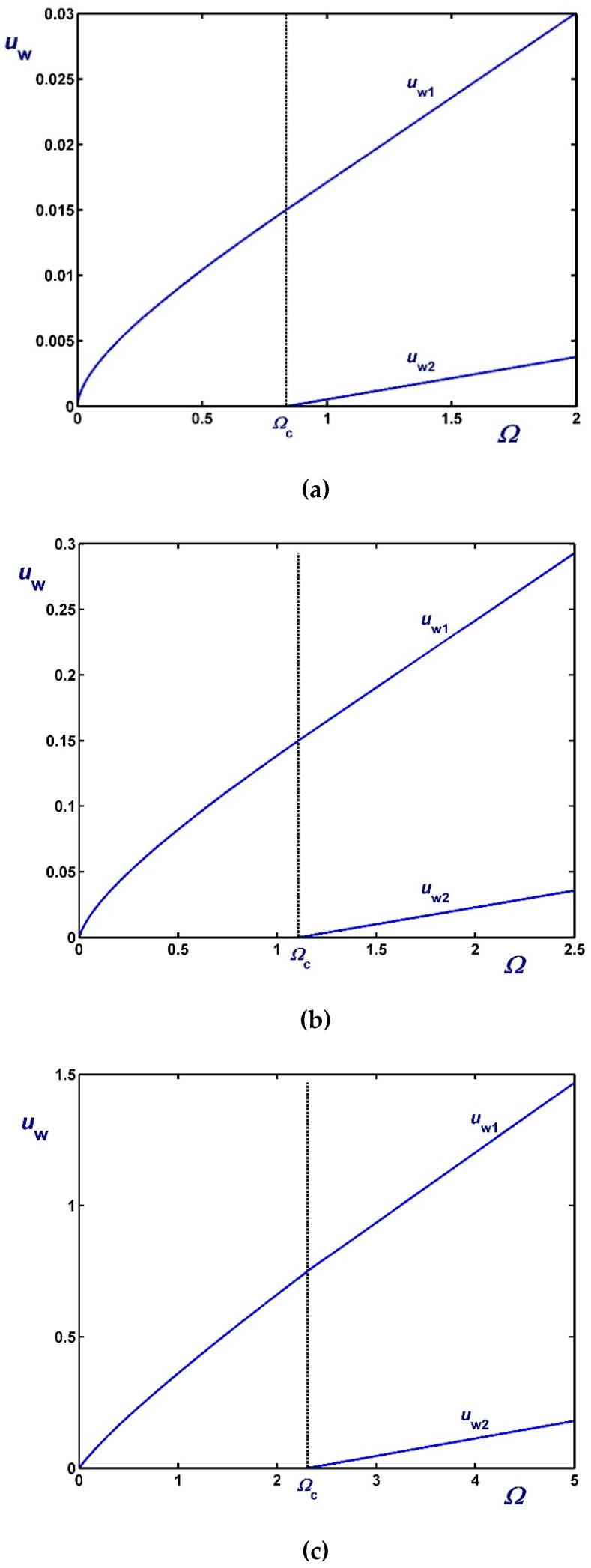
Slip velocities for κ=0.5 and different slip numbers with non-zero slip yield stress $\tau_{c}^{*}=\tau_{0}^{*}$ : (**a**) $B_{1}=B_{2}=0.01$ with $\Omega_{c}=0.083685$ (weak slip); (**b**) $B_{1}=B_{2}=0.1$ with $\Omega_{c}=1.10685$ (moderate slip); (c) $B_{1}=B_{2}=0.5$ with $\Omega_{c}=2.30685$ (strong slip).

**Figure 11:**
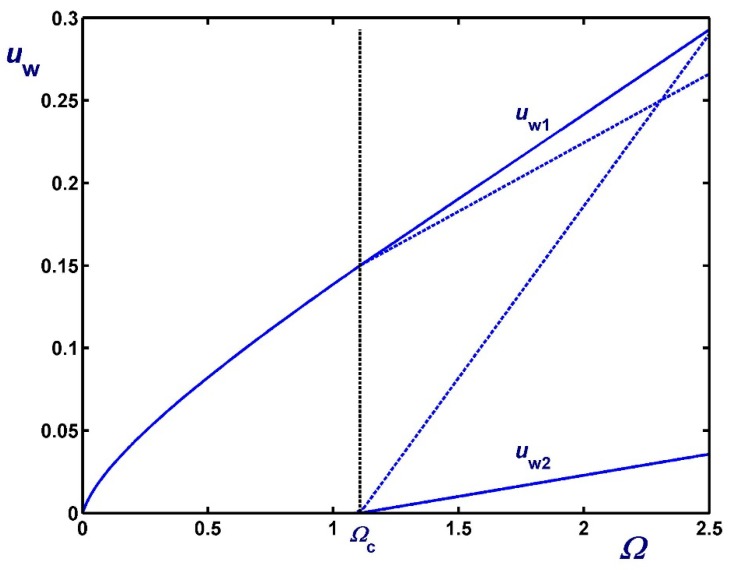
Slip velocities for κ=0.5, $B_{1}=0.1$ and $B_{2}=0.1$ (moderate slip along both cylinders, solid) and $B_{2}=1$ (strong slip along the outer cylinder, dashed) in the case of non-zero slip yield stress $\tau_{c}^{*}=\tau_{0}^{*}$. The critical angular velocity is $\Omega_{c}=1.10685$.

**Figure 12:**
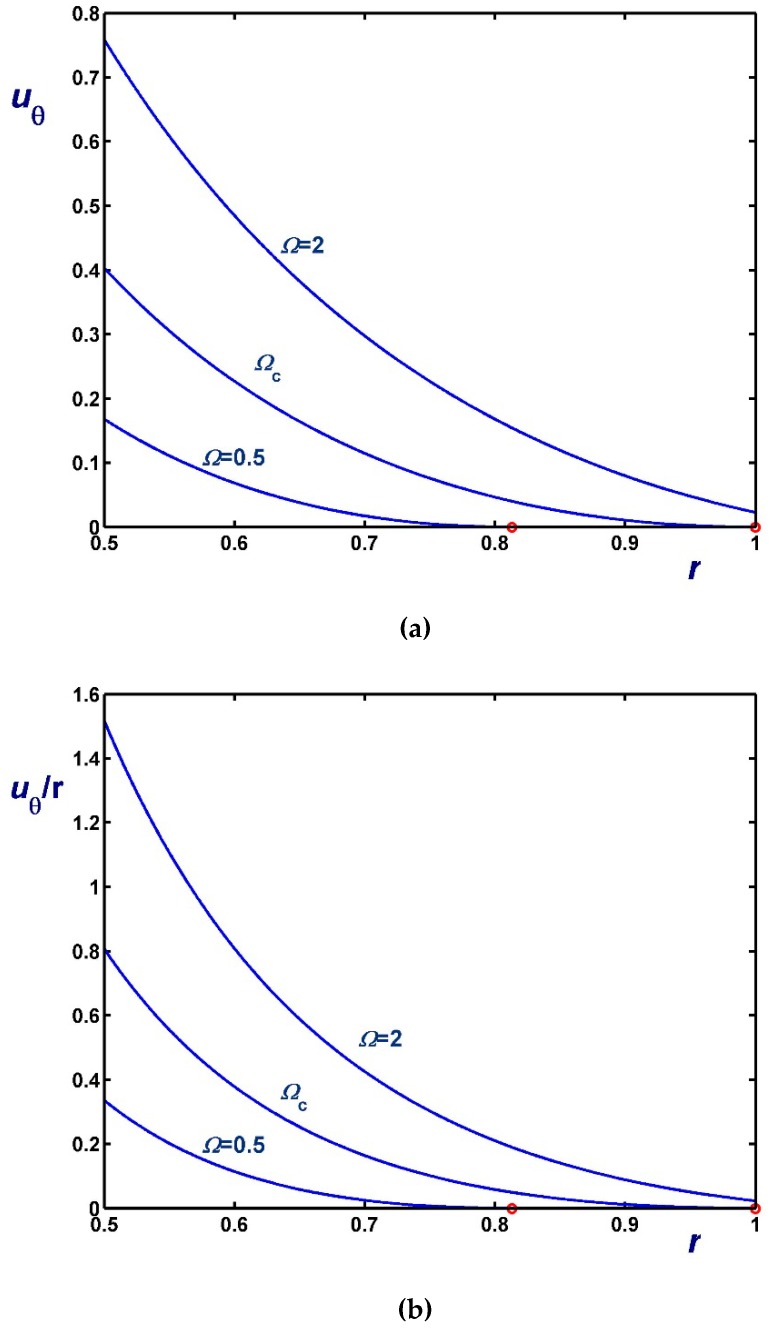
Velocity profiles in circular Couette flow of a Bingham plastic with non-zero slip yield stress $\tau_{c}^{*}=\tau_{0}^{*}$, κ=0.5 and $B_{1}=B_{2}=0.1$ (moderate slip); (**a**) azimuthal velocity; (**b**) angular velocity; $\Omega_{c}=1.10685$. The red circles indicate the yield points for the velocity profiles corresponding to Regime I.

**Figure 13:**
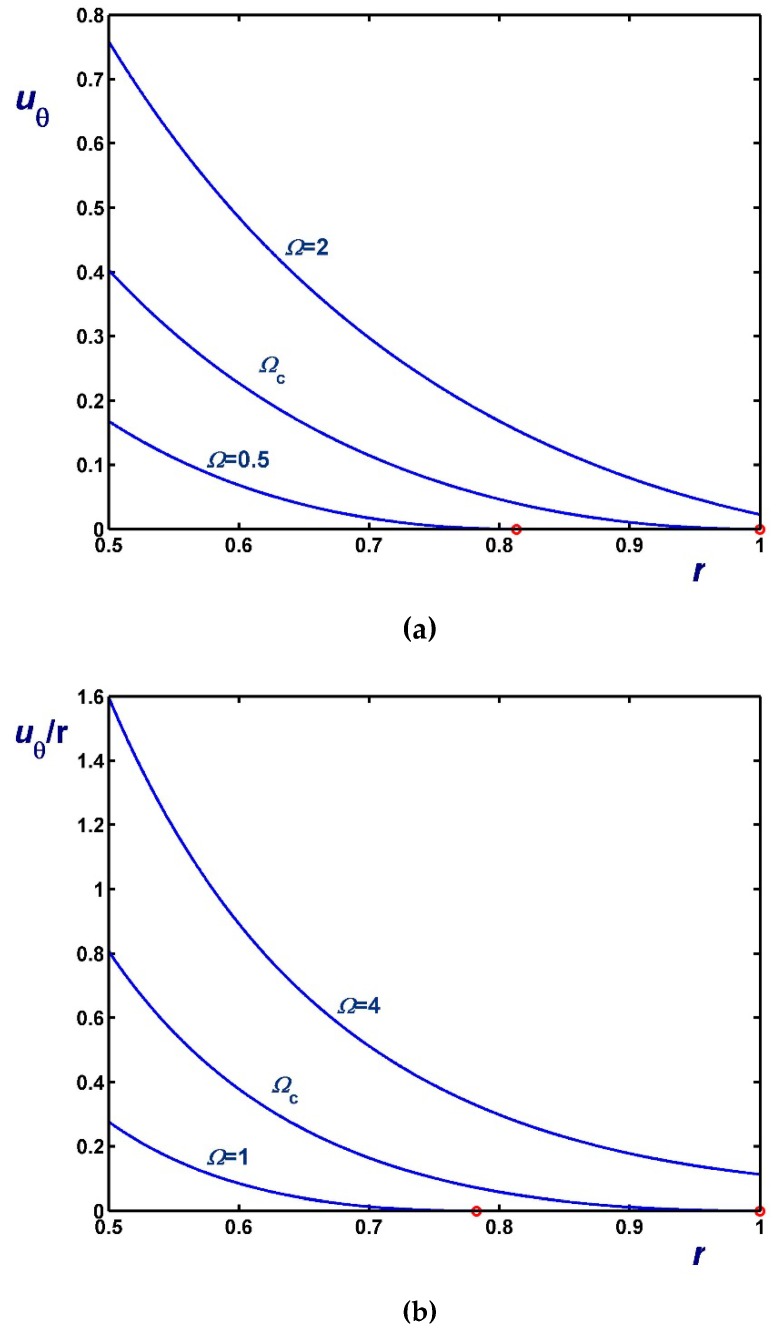
Velocity profiles in circular Couette flow of a Bingham plastic with non-zero slip yield stress $\tau_{c}^{*}=\tau_{0}^{*}$, κ=0.5 and $B_{1}=B_{2}=0.5$ (strong slip); (**a**) azimuthal velocity; (**b**) angular velocity; $\Omega_{c}=1.10685$. The red circles indicate the yield points for the velocity profiles corresponding to Regime I.

Figure 10 illustrates the variation of the slip velocities with Ω for κ=0.5 and three values of the slip numbers which are taken to be equal, i.e., $B_{1}=B_{2}=0.01$ (weak slip), $B_{1}=B_{2}=0.1$ (moderate slip) and $B_{1}=B_{2}=0.5$ (strong slip). The effect of $B_{2}$ on the two slip velocities is demonstrated in Figure 11 where κ=0.5 and $B_{1}=0.1$. As dictated by Equations (61) and (62), both slip velocities vary linearly with Ω. As $B_{2}$ is increased the rate of change of $u_{w2}$ increases unlike that of $u_{w1}$ and for certain choices of Ω and $B_{2}$, $u_{w2}$ may be greater than $u_{w1}$. In Figure 12, we plotted the velocity profiles for κ=0.5, $B_{1}=B_{2}=0.1$ and Ω=0.5 (Regime I), $\Omega =\Omega_{c}=1.10685$, and Ω=2 (Regime II). Similar results are presented in Figure 13 for much stronger slip with $B_{1}=B_{2}=0.5$. 

### 3.2. The Case $\tau_{c}^{*}<\tau_{0}^{*}$

For the sake of simplicity, it is assumed here that the slip yield stress and the slip coefficient are the same at both walls:

(64) $$u_{wi}^{*}=\{\begin{array}{ll} 0, & \tau_{wi}^{*}\leq \tau_{c}^{*}\\ \frac{\tau_{wi}^{*}-\tau_{c}^{*}}{\beta^{*}}, & \tau_{wi}^{*}>\tau_{c}^{*} \end{array},\;i=1,2.$$

**Figure 14:**
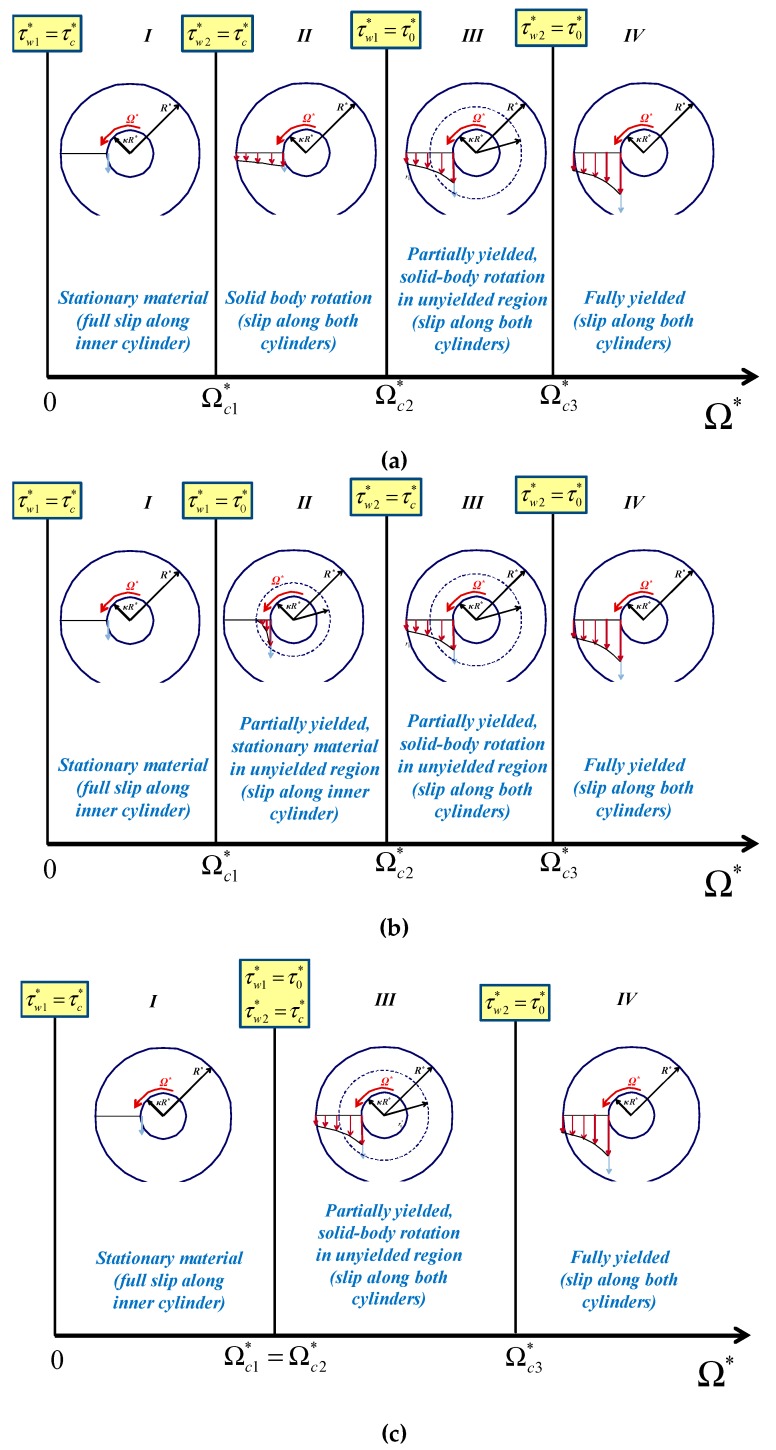
Flow regimes in circular Couette flow of a Bingham plastic when the inner cylinder is rotating with non-zero slip yield stress such that $\tau_{c}^{*}<\tau_{0}^{*}$: (**a**) $\tau_{c}^{*}<\kappa^{2}\tau_{0}^{*}$; (**b**) $\tau_{c}^{*}>\kappa^{2}\tau_{0}^{*}$; (**c**) $\tau_{c}^{*}=\kappa^{2}\tau_{0}^{*}$ (the second regime is not observed). It should be noted that $\tau_{w2}^{*}=\tau_{0}^{*}$ is equivalent to $\tau_{w1}^{*}=\tau_{0}^{*}/\kappa^{2}$.

Since $\tau_{c}^{*}<\tau_{0}^{*}$, it is possible to rotate the inner cylinder when $\tau_{w1}^{*}>\tau_{c}^{*}$ while the material remains stationary, which simply implies that the material slips along the rotating cylinder. As illustrated in Figure 14, three different scenarios are possible, which correspond to the cases $\tau_{c}^{*}<\kappa^{2}\tau_{0}^{*}$, $\tau_{c}^{*}>\kappa^{2}\tau_{0}^{*}$, and $\tau_{c}^{*}=\kappa^{2}\tau_{0}^{*}$ discussed below. 

When $\tau_{c}^{*}<\kappa^{2}\tau_{0}^{*}$, four flow regimes are observed, defined by three critical angular velocities $\Omega_{c1}^{*}$, $\Omega_{c2}^{*}$ and $\Omega_{c3}^{*}$ corresponding to $\tau_{w2}^{*}=\tau_{c}^{*}$, $\tau_{w1}^{*}=\tau_{0}^{*}$, and $\tau_{w2}^{*}=\tau_{0}^{*}$, respectively. The derivation of the solution is along the same lines as in Section 2. As above, the dimensionless form of the solution is provided. The three critical angular velocities are given by

(65) $$\Omega_{c1}=\left(\frac{1}{\kappa^{2}}-1\right)B\tau_{c},$$

(66) $$\Omega_{c2}=(1+\kappa )B\left(1-\kappa +\kappa^{2}-\tau_{c}\right)$$

and

(67) $$\Omega_{c3}=\frac{1+2(1+\kappa^{3})B}{2\kappa^{2}}-(1+\kappa )B\tau_{c}-\ln\frac{1}{\kappa }-\frac{1}{2},$$

where $B\equiv \mu^{*}/(\beta^{*}\kappa R^{*})$ and $\tau_{c}\equiv \tau_{c}^{*}/\tau_{0}^{*}$. It should be noted that in the limit of $\tau_{c}\to 0$, $\Omega_{c1}$ also vanishes while $\Omega_{c2}$ and $\Omega_{c3}$ are reduced to the two critical angular velocities for the Navier-slip case, i.e., Equations (44) and (45) (with $B_{1}=B_{2}=B$).

In Regime I ($0<\Omega \leq \Omega_{c1}$), the material slips along the rotating cylinder remaining thus stationary ($u_{\theta }=0$). The wall shear stress and the inner slip velocity are given by:

(68) $$\tau_{w1}=\tau_{c}+\frac{\Omega }{B}$$

and

(69) $$u_{w1}=\kappa \Omega$$

In Regime II ($\Omega_{c1}<\Omega \leq \Omega_{c2}$), the material slips along both walls and rotates as a solid body at an angular velocity smaller than Ω:

(70) $$u_{\theta }(r)=\frac{\kappa^{3}}{1+\kappa^{3}}\left[\Omega -\left(\frac{1}{\kappa^{2}}-1\right)B\tau_{c}\right]r.$$

The wall shear stresses and the two slip velocities are given by

(71) $$\tau_{w1}=\frac{1}{1+\kappa^{3}}\left[\frac{\Omega }{B}+(1+\kappa )\tau_{c}\right],\;\tau_{w2}=\kappa^{2}\tau_{w1}$$

and

(72) $$u_{w1}=\frac{\kappa }{1+\kappa^{3}}\left[\Omega +\kappa (1-\kappa^{2})B\tau_{c}\right],\;u_{w2}=\frac{1}{1+\kappa^{3}}\left[\kappa^{3}\Omega -\kappa (1-\kappa^{2})B\tau_{c}\right].$$

In Regime III ($\Omega_{c2}<\Omega \leq \Omega_{c3}$), the material is partially yielded, rotating as a solid body in the unyielded region and exhibiting slip along both walls. Hence, the velocity has two branches as follows:

(73) $$u_{\theta }(r)=r\{\begin{array}{cc} \Omega +\ln\frac{r}{\kappa }+B\tau_{c}-\frac{r_{0}^{2}}{2\kappa^{2}}\left(1+2B-\frac{\kappa^{2}}{r^{2}}\right), & \kappa \leq r\leq r_{0}\\ \kappa B(r_{0}^{2}-\tau_{c}), & r_{0}<r\leq 1 \end{array},$$

where the yield point is the root of

(74) $$\Omega +\ln\frac{r_{0}}{\kappa }-\left[1+2(1+\kappa^{3})B\right]\frac{r_{0}^{2}}{2\kappa^{2}}+(1+\kappa )B\tau_{c}+\frac{1}{2}=0.$$

The wall shear stresses and the two slip velocities are given by

(75) $$\tau_{w1}=\frac{r_{0}^{2}}{\kappa^{2}},\;\tau_{w2}=r_{0}^{2}$$

and

(76) $$u_{w1}=\kappa B\left(\frac{r_{0}^{2}}{\kappa^{2}}-\tau_{c}\right),\;u_{w2}=\kappa B\left(r_{0}^{2}-\tau_{c}\right).$$

Finally, in Regime IV ($\Omega >\Omega_{c3}$), the material is fully yielded exhibiting slip along both walls:

(77) $$u_{\theta }(r)=r\left[\Omega +\ln\frac{r}{\kappa }+B\tau_{c}-\frac{\Omega +\ln\frac{1}{\kappa }+(1+\kappa )B\tau_{c}}{1-\kappa^{2}+2(1+\kappa^{3})B}\left(1+2B-\frac{\kappa^{2}}{r^{2}}\right)\right],$$

(78) $$\tau_{w1}=\frac{2\left[\Omega +\ln\frac{1}{\kappa }+(1+\kappa )B\tau_{c}\right]}{1-\kappa^{2}+2(1+\kappa^{3})B},\;\tau_{w2}=\kappa^{2}\tau_{w1},$$

(79) $$u_{w1}=\frac{\kappa B\left[2\left(\Omega +\ln\frac{1}{\kappa }\right)-(1-\kappa^{2})(1-2\kappa B)\tau_{c}\right]}{1-\kappa^{2}+2(1+\kappa^{3})B}$$

and

(80) $$u_{w2}=\frac{\kappa B\left[2\kappa^{2}\left(\Omega +\ln\frac{1}{\kappa }\right)-(1-\kappa^{2})(1+2B)\tau_{c}\right]}{1-\kappa^{2}+2(1+\kappa^{3})B}.$$

Four regimes are also encountered when $\tau_{c}^{*}>\kappa^{2}\tau_{0}^{*}$ (Figure 14b); the three critical values of the angular velocity defining these regimes now correspond to $\tau_{w1}^{*}=\tau_{0}^{*}$, $\tau_{w2}^{*}=\tau_{c}^{*}$, and $\tau_{w2}^{*}=\tau_{0}^{*}$. Hence, $\Omega_{c3}$ remains the same as above. In fact, the solution remains the same as that for $\tau_{c}^{*}<\kappa^{2}\tau_{0}^{*}$ in Regimes I, III, and IV; only the critical angular velocities $\Omega_{c1}$ and $\Omega_{c2}$ and the solution in Regime II are different. The first two critical angular velocities now read:

(81) $$\Omega_{c1}=(1-\tau_{c})B$$

and

(82) $$\Omega_{c2}=\frac{[1+2(1-\kappa^{2})B]\tau_{c}}{2\kappa^{2}}-\ln\frac{\sqrt{\tau_{c}}}{\kappa }-\frac{1}{2}.$$

In Regime II, the material is partially yielded and remains stationary in the unyielded region. Thus, the velocity is given by

(83) $$u_{\theta }(r)=r\{\begin{array}{cc} \Omega +\ln\frac{r}{\kappa }+B\tau_{c}-\frac{r_{0}^{2}}{2\kappa^{2}}\left(1+2B-\frac{\kappa^{2}}{r^{2}}\right), & \kappa \leq r\leq r_{0}\\ 0, & r_{0}<r\leq 1 \end{array},$$

where the yield radius $r_{0}$ is now the solution of

(84) $$\Omega +\ln\frac{r_{0}}{\kappa }-(1+2B)\frac{r_{0}^{2}}{2\kappa^{2}}+B\tau_{c}+\frac{1}{2}=0.$$

The inner wall shear stress and the corresponding slip velocity are:

(85) $$\tau_{w1}=\frac{r_{0}^{2}}{\kappa^{2}}$$

and

(86) $$u_{w1}=\kappa B\left(\frac{r_{0}^{2}}{\kappa^{2}}-\tau_{c}\right).$$

Note that the flow is still partially yielded in Regime III, but the unyielded material now rotates as a solid body. It turns out that the critical yield radius corresponding to $\Omega_{c2}$ is $r_{0c}=\sqrt{\tau_{c}}$.

In the special case where $\tau_{c}^{*}=\kappa^{2}\tau_{0}^{*}$, the two critical angular velocities $\Omega_{c1}$ and $\Omega_{c2}$ coincide. Indeed, substituting $\tau_{c}=\kappa^{2}$ to Equations (65), (66), (81) and (82) yields

(87) $$\Omega_{c1}=\Omega_{c2}=(1-\kappa^{2})B.$$

in all cases. Hence, only Regimes I, III, and IV are observed, and the expressions presented above apply. 

**Figure 15:**
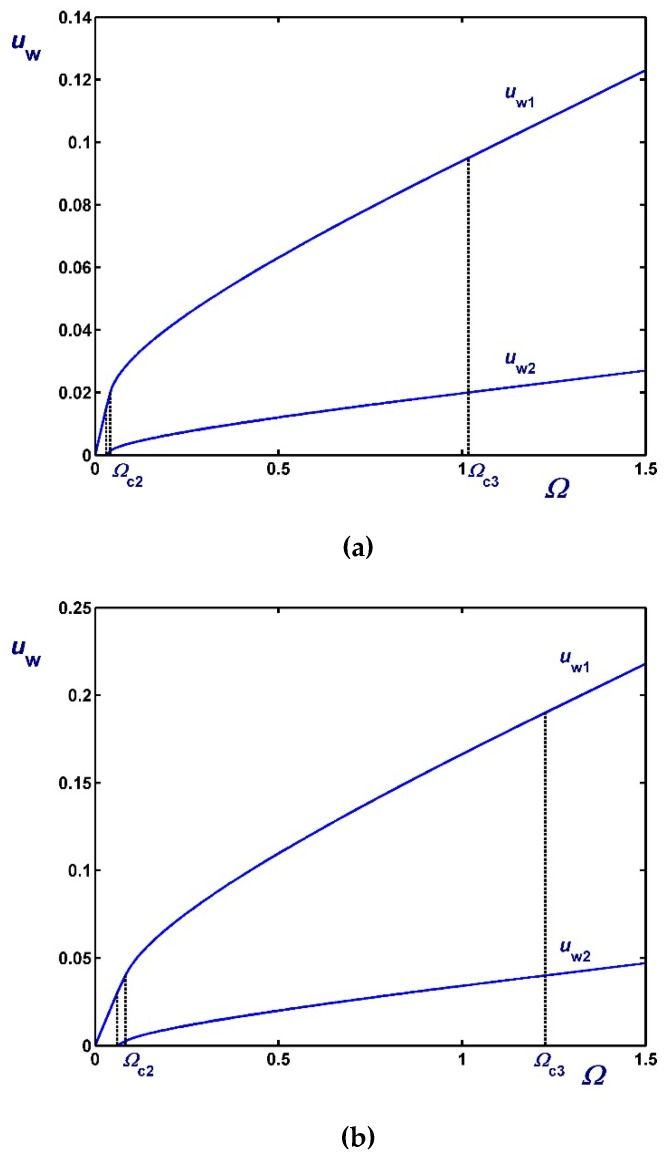

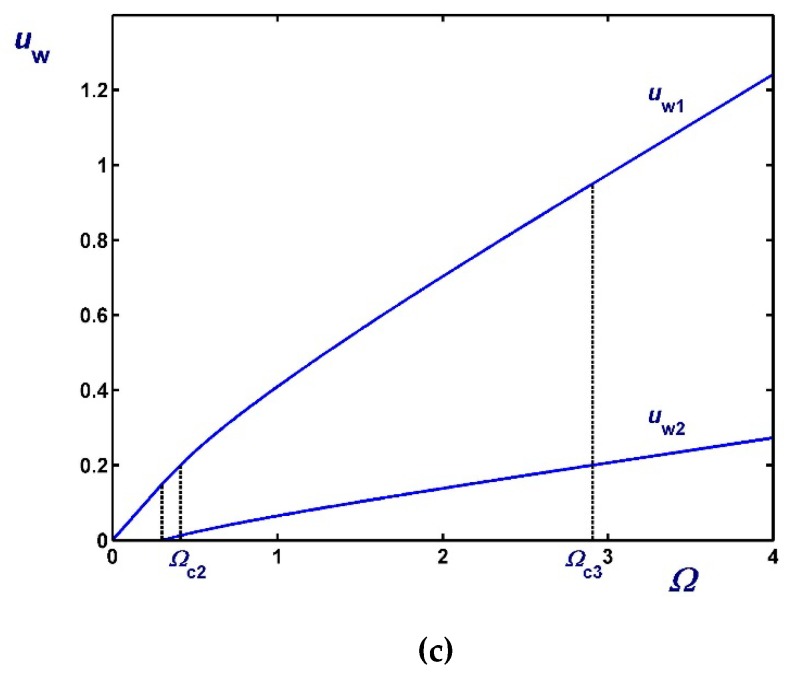
Slip velocities for κ=0.5, $\tau_{c}=0.2$ and different slip numbers: (**a**) B=0.05 (weak slip); (**b**) B=0.1 (moderate slip); (**c**) B=0.5 (strong slip).

**Figure 16:**
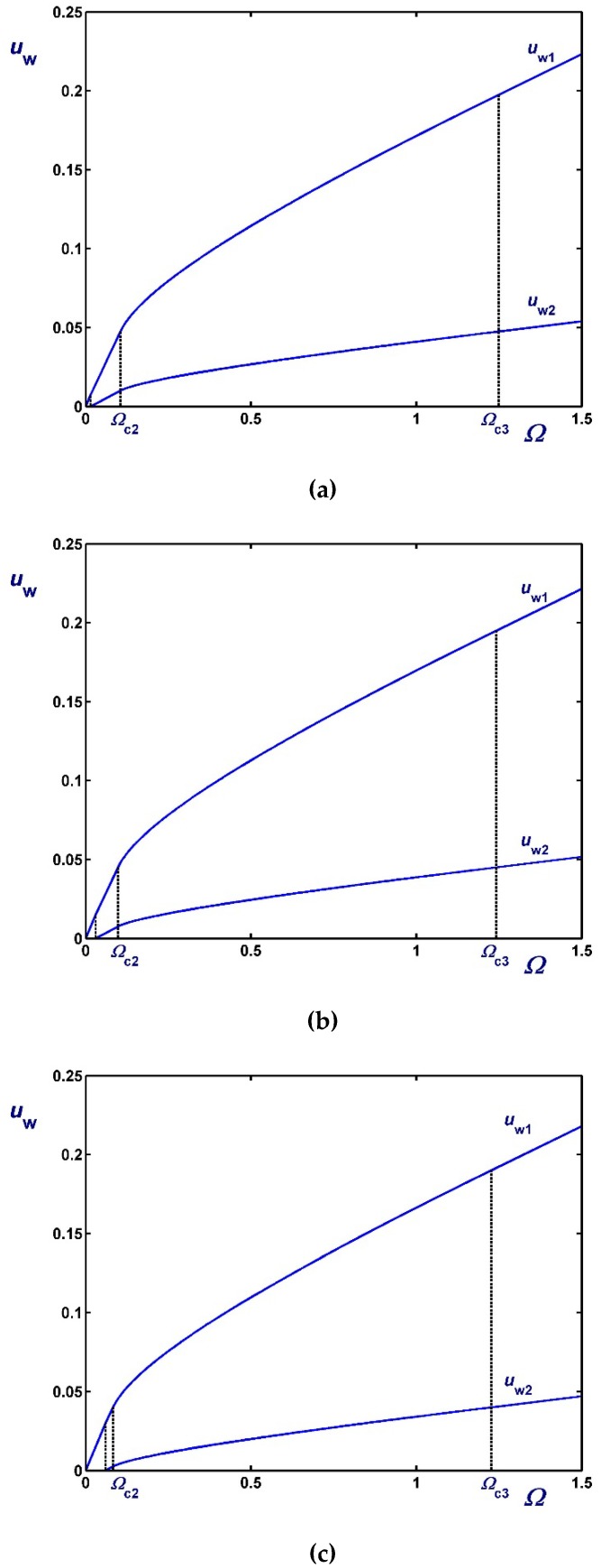
Slip velocities for κ=0.5, B=0.1 and different values of the dimensionless slip yield stress number: (**a**) $\tau_{c}=0.05$; (**b**) $\tau_{c}=0.1$; (**c**) $\tau_{c}=0.2$.

**Figure 17:**
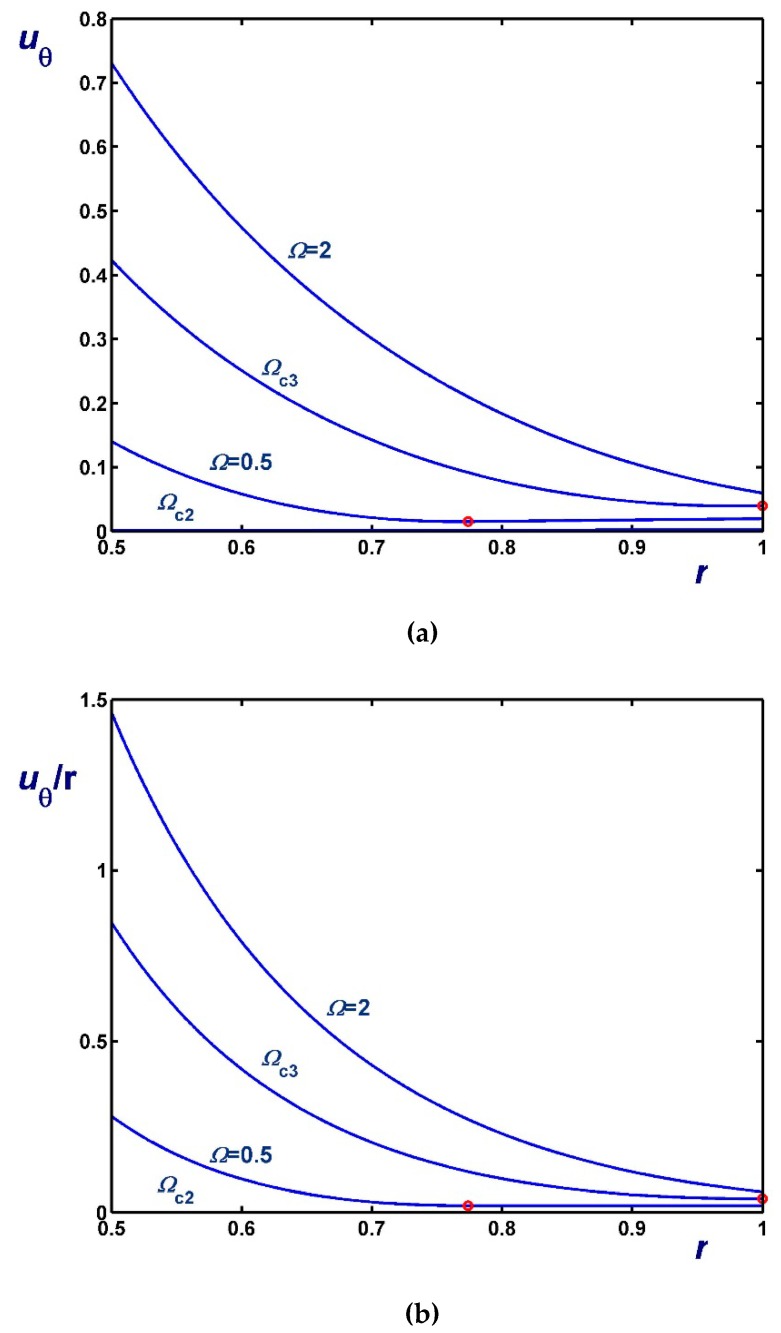
Velocity profiles in circular Couette flow of a Bingham plastic with non-zero slip yield stress $\tau_{c}=0.2$, κ=0.5 and B=0.1 (moderate slip); (**a**) azimuthal velocity; (**b**) angular velocity; $\Omega_{c1}=0.06$, $\Omega_{c2}=0.0825$, $\Omega_{c3}=1.22685$. The red circles indicate the yield points for the velocity profiles corresponding to Regime III. The profiles for $\Omega =\Omega_{c2}$ essentially coincide with the *x*-axis.

As dictated by the above solutions, the slip velocities vary linearly with the angular velocity Ω in all regimes but Regime III, i.e., when the material is partially yielded. This is illustrated in Figure 15 where results are shown for κ=0.5, $\tau_{c}=0.2$ (i.e., $\tau_{c}<\kappa^{2}$) and different slip numbers corresponding to weak, moderate, and strong slip. Note that all the critical angular velocities and the two slip velocities increase with B. The effect of the dimensionless slip yield stress $\tau_{c}$ is shown in Figure 16, where the slip number is now fixed (B=0.1) and results for $\tau_{c}=0.05$, 0.1 and 0.2 are shown. As expected, slip velocities are reduced with $\tau_{c}$. It can also be observed that $\Omega_{c2}$ and $\Omega_{c3}$ decrease with $\tau_{c}$, which is also obvious from Equations (66) and (67). As $\tau_{c}$ tends to $\kappa^{2}$ both $\Omega_{c1}$ and $\Omega_{c2}$ tend to $(1-\kappa^{2})B$, as predicted by Equation (87). Finally, in Figure 17 we show the profiles of the angular and azimuthal velocities for κ=0.5, $\tau_{c}=0.2$, B=0.1 and various values of the angular velocity Ω. Note that the profiles for $\Omega =\Omega_{c2}$ essentially coincide with the *x*-axis. 

## 4. Conclusions

We have systematically studied the Couette flow of a Bingham plastic in the presence of Navier slip and in the case where slip occurs only above a non-zero slip yield stress. All flow regimes have been identified and the corresponding critical angular velocities have been determined. 

The solutions presented here may be useful in assessing slip effects in Couette experiments on viscoplastic materials and the implications of calibrating a Couette rheometer with “standard fluids” under the assumption of no slip. Our current research plans include the numerical solution of the flow of a Herschel-Bulkley fluid in a Couette rheometer in both steady-state and time-dependent settings.
